# Feeding and Fasting Signals Converge on the LKB1-SIK3 Pathway to Regulate Lipid Metabolism in *Drosophila*

**DOI:** 10.1371/journal.pgen.1005263

**Published:** 2015-05-21

**Authors:** Sekyu Choi, Dae-Sik Lim, Jongkyeong Chung

**Affiliations:** 1 Department of Biological Sciences, Korea Advanced Institute of Science and Technology, Daejon, Republic of Korea; 2 National Creative Research Initiatives Center for Energy Homeostasis Regulation, Seoul National University, Seoul, Republic of Korea; 3 Institute of Molecular Biology and Genetics, Seoul National University, Seoul, Republic of Korea; 4 National Creative Research Initiatives Center for Cell Division and Differentiation, Korea Advanced Institute of Science and Technology, Daejon, Republic of Korea; 5 School of Biological Sciences, Seoul National University, Seoul, Republic of Korea; Washington University Medical School, UNITED STATES

## Abstract

LKB1 plays important roles in governing energy homeostasis by regulating AMP-activated protein kinase (AMPK) and other AMPK-related kinases, including the salt-inducible kinases (SIKs). However, the roles and regulation of LKB1 in lipid metabolism are poorly understood. Here we show that *Drosophila* LKB1 mutants display decreased lipid storage and increased gene expression of *brummer*, the *Drosophila* homolog of adipose triglyceride lipase (ATGL). These phenotypes are consistent with those of SIK3 mutants and are rescued by expression of constitutively active SIK3 in the fat body, suggesting that SIK3 is a key downstream kinase of LKB1. Using genetic and biochemical analyses, we identify HDAC4, a class IIa histone deacetylase, as a lipolytic target of the LKB1-SIK3 pathway. Interestingly, we found that the LKB1-SIK3-HDAC4 signaling axis is modulated by dietary conditions. In short-term fasting, the adipokinetic hormone (AKH) pathway, related to the mammalian glucagon pathway, inhibits the kinase activity of LKB1 as shown by decreased SIK3 Thr196 phosphorylation, and consequently induces HDAC4 nuclear localization and *brummer* gene expression. However, under prolonged fasting conditions, AKH-independent signaling decreases the activity of the LKB1-SIK3 pathway to induce lipolytic responses. We also identify that the *Drosophila* insulin-like peptides (DILPs) pathway, related to mammalian insulin pathway, regulates SIK3 activity in feeding conditions independently of increasing LKB1 kinase activity. Overall, these data suggest that fasting stimuli specifically control the kinase activity of LKB1 and establish the LKB1-SIK3 pathway as a converging point between feeding and fasting signals to control lipid homeostasis in *Drosophila*.

## Author Summary

Liver kinase B1 (LKB1), a serine/threonine kinase, controls 14 different AMP-activated protein kinase (AMPK) family kinases, including salt-inducible kinase 3 (SIK3), suggesting that it plays a variety of roles. Using the fruit fly as an *in vivo* model system, we reveal that LKB1 kinase activity is critical for lipid storage and controls the lipolysis pathway in the fat body, which is equivalent to mammalian adipose and liver tissue. We find that the lipolytic defects of LKB1 mutants are rescued by the expression of constitutively active SIK3 in the fat body. We show that LKB1 and SIK3 regulate lipid storage by altering the gene expression of *brummer*, the *Drosophila* homolog of human adipose triglyceride lipase (ATGL), a critical lipolytic gene. We also identify that LKB1-SIK3 signaling controls the nuclear and cytosolic localization of the class IIa deacetylase HDAC4 via SIK3-dependent phosphorylation in feeding and fasting conditions, respectively. Collectively, these data suggest that the LKB1-SIK3-HDAC4 pathway plays a critical role in maintaining fly lipid homeostasis in response to dietary conditions.

## Introduction

Perturbation of energy homeostasis either directly or indirectly causes human health problems such as obesity and type II diabetes [1]. Lipid stores are the major energy reserves in animals and are dynamically regulated by alternating between the lipogenesis and lipolysis cycles in response to food availability. Dissecting the regulatory mechanisms of lipid homeostasis is therefore essential to our understanding of how energy metabolism is maintained.

*Drosophila* has emerged as a useful genetic model organism for studying lipid homeostasis and energy metabolism [2]. *Drosophila* lipid reserves are mainly stored as triacylglycerol (TAG) in the fat body, the insect equivalent of mammalian adipose tissue. In addition, lipolytic factors are evolutionarily conserved between insects and mammals. Brummer (Bmm) is the *Drosophila* homolog of ATGL, a key regulator of lipolysis. *bmm* mutant flies are obese and display partial defects in lipid mobilization [3]. Furthermore, hormonal regulation of lipid metabolism is also highly conserved in *Drosophila*. Under starvation conditions, the primary role of AKH, the functional analogue of glucagon and β-adrenergic signaling in mammals [4,5], is to stimulate lipid mobilization by activating AKH receptor (AKHR) [6] and consequently inducing cAMP/PKA signaling in the fat body [7]. A report demonstrated that AKH acts in parallel with Bmm to regulate lipolysis and that AKHR mutation leads to obesity phenotypes and defects in fat mobilization [7]. However, *bmm* expression is hyperstimulated in starved AKHR mutants [7], implying the existence of an unknown regulatory mechanism between Bmm and AKHR in *Drosophila*.

LKB1 (liver kinase B1, also known as STK11) is a serine/threonine kinase that was first identified as a tumor suppressor gene associated with Peutz-Jeghers syndrome [8,9]. LKB1 phosphorylates and activates AMP-activated protein kinase (AMPK) in response to cellular energy status, thus controlling cell metabolism, cell structures, apoptosis, etc. [10–13]. Moreover, LKB1 is the master upstream protein kinase for 12 AMPK-related kinases, including salt-inducible kinases (SIKs) [14], suggesting that it plays diverse roles. Although the metabolic functions of AMPK have been highly studied, the *in vivo* functions of LKB1 and AMPK-related kinases in metabolism, including lipid homeostasis, are still largely unknown [15]. Recent reports showed that LKB1 is required for the growth and differentiation of white adipose tissue [16] and that SIK3 maintains lipid storage size in adipose tissues [17]. In addition, we and others determined that *Drosophila* SIK family kinases regulate lipid levels and starvation responses [18,19]. However, to further understand the roles and mechanisms of LKB1 signaling in lipid metabolism, proper genetic animal models are urgently required.

Here we demonstrate the role of LKB1 and its downstream SIK3 in the regulation of lipid homeostasis using *Drosophila* as an *in vivo* model system. We demonstrated that LKB1-activated SIK3 regulates the nucleocytoplasmic localization of HDAC4 to control lipolytic gene expression. We also identified that DILPs modulate SIK3 activity via Akt-dependent phosphorylation and the AKH pathway regulates LKB1 activity in phosphorylating SIK3 to control its lipolytic responses upon short-term fasting. Furthermore, we identified that AKH-independent signaling modulates the LKB1-SIK3-HDAC4 pathway upon prolonged fasting. Altogether, these studies showed that the LKB1-SIK3 signaling pathway plays a crucial regulatory role in maintaining lipid homeostasis in *Drosophila*.

## Results and Discussion

### *Drosophila LKB1* mutants display reduced lipid storage in the fat body

**Fig 1:**
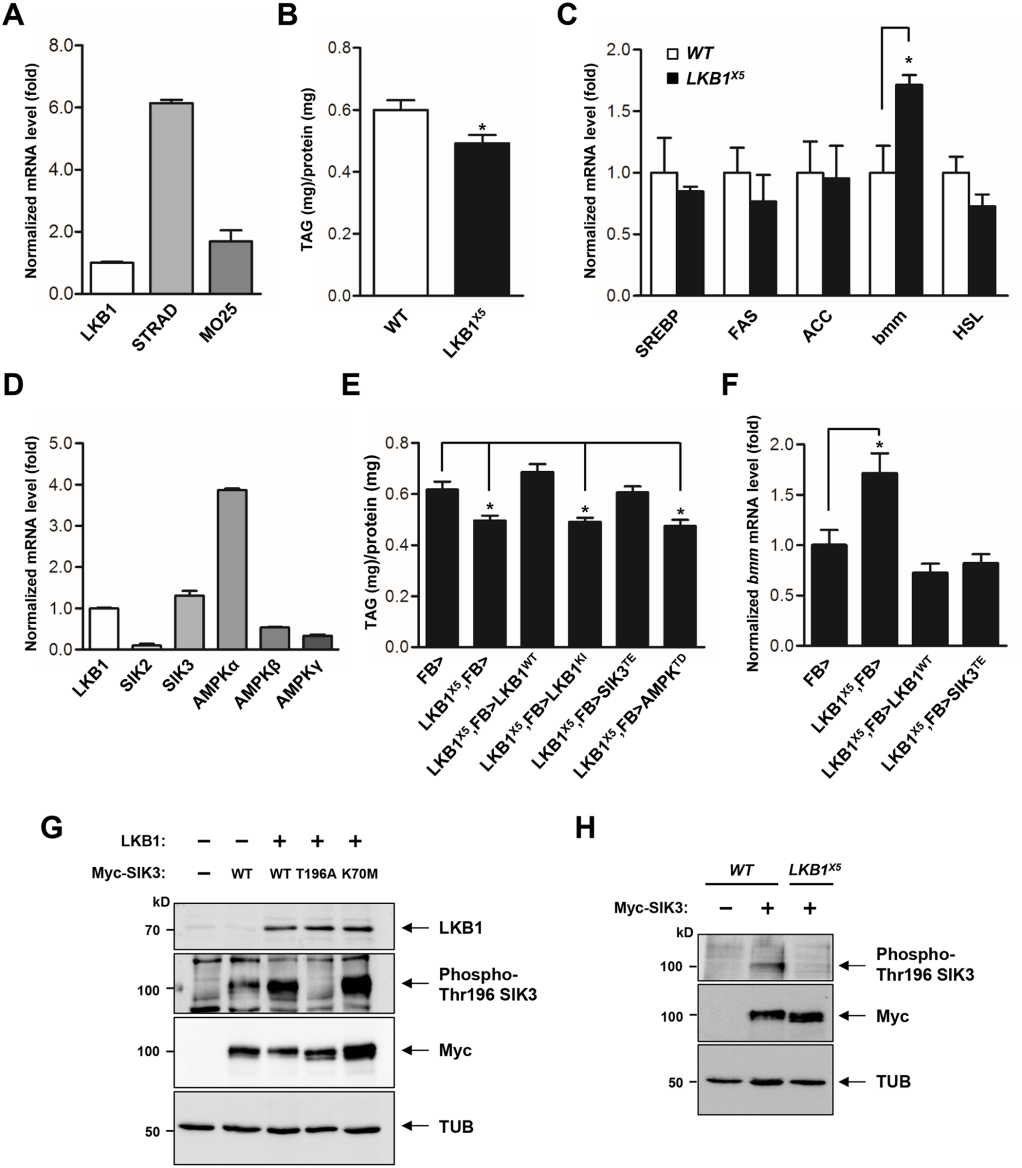
LKB1 and its downstream kinase SIK3 are required for lipid homeostasis. (A) qPCR analysis of LKB1 and its cofactors required for the catalytic activity, STRAD and MO25, in *Drosophila* larvae under feeding condition. (B) TAG amounts of wild-type and *LKB1* mutant larvae (*n* = 10 per genotype). (C) qPCR analysis for lipogenic genes (*SREBP*, *FAS* and *ACC*) and lipolytic genes (*bmm* and *HSL*) in wild-type and *LKB1* mutant larvae at mid-to-late L2 (60 hr AEL) stage under feeding conditions. (D) qPCR analysis of LKB1, SIKs (SIK2 and SIK3), and AMPK complex (AMPKα, AMPKβ, and AMPKγ) in larvae. (E) TAG amounts in LKB1 mutants following fat body-specific expression of wild-type, kinase-dead (K201I) LKB1, constitutively active (T196E) SIK3 or constitutively active (T184D) AMPK. Genotypes are as follows: FB> (*FB-Gal4*/+), LKB1^X5^,FB> (*FB-Gal4/+;LKB1* ^*X5*^ */LKB1* ^*X5*^), LKB1^X5^,FB>LKB1^WT^ (*FB-Gal4/UAS-LKB1;LKB1* ^*X5*^ */LKB1* ^*X5*^), LKB1^X5^,FB>LKB1^KI^ (*FB-Gal4/UAS-LKB1 K201I;LKB1* ^*X5*^ */LKB1* ^*X5*^), LKB1^X5^,FB>SIK3^TE^ (*FB-Gal4/UAS-SIK3 T196E;LKB1* ^*X5*^ */LKB1* ^*X5*^), and LKB1^X5^,FB>AMPK^TD^ (*FB-Gal4/UAS-AMPK T184D;LKB1* ^*X5*^ */LKB1* ^*X5*^) (*n* = 10 per genotype). (F) qPCR analysis of *bmm* gene expression in *LKB1* mutants following fat body-specific expression of wild-type LKB1 or constitutively active (T196E) SIK3 at mid-to-late L2 stage under feeding condition. Genotypes are as follows: FB> (*FB-Gal4*/+), LKB1^X5^,FB> (*FB-Gal4/+;LKB1* ^*X5*^ */LKB1* ^*X5*^), LKB1^X5^,FB>LKB1^WT^ (*FB-Gal4/UAS-LKB1;LKB1* ^*X5*^ */LKB1* ^*X5*^), and LKB1^X5^,FB>SIK3^TE^ (*FB-Gal4/UAS-SIK3 T196E;LKB1* ^*X5*^ */LKB1* ^*X5*^). (G) Immunoblot analyses showing the effect of LKB1 on Thr196 phosphorylation of SIK3 protein in larvae. Wild-type and kinase-dead (K70M) SIK3 were highly phosphorylated at Thr196 by LKB1 (second panel). SIK3^T196A^ was used as a control. *FB-Gal4* was used to drive transgene expression in the fat body. (H) Immunoblot analyses showing relative amounts of SIK3 Thr196 phosphorylation in wild-type and *LKB1* ^*X5*^ mutant larvae. The phosphorylation was absolutely dependent on LKB1 (first panel). *FB-Gal4* was used to drive transgene expression. (G-H) Anti-LKB1, -phospho-Thr196 SIK3, -Myc (SIK3 protein), and -β-tubulin (TUB) antibodies were used. Data are presented as mean ± SEM (*P < 0.05).

LKB1 functions in a complex with two scaffolding proteins, STE20-related adaptor (STRAD) and mouse protein 25 (MO25) [20,21]. As the first step toward elucidation of the role of LKB1 in lipid metabolism, we demonstrated the gene expression of each component of the LKB1 complex in the fat body (Fig 1A), suggesting that *Drosophila* LKB1 forms the heterotrimeric complex when activated in tissues. Additionally, we characterized an *LKB1*-null mutant line, *LKB1* ^*X5*^ [22], and found that these flies showed markedly decreased lipid storage compared to wild-type flies, despite having similar food intake and retaining expression of the lipogenic genes (SREBP, FAS, and ACC) (Figs 1B, 1C and S1A). However, expression of *bmm* and lipolysis activity were elevated in *LKB1* ^*X5*^ mutants (Figs 1C and S1B, respectively). Moreover, transgenic expression of wild-type LKB1 with two different fat body drivers (*FB-Gal4* and *cg-Gal4*) rescued the decreased lipid levels and increased *bmm* expression phenotypes of *LKB1* ^*X5*^ mutants, whereas expression of the kinase-dead form of LKB1 (LKB1 K201I) did not (Figs 1E, 1F, S2A and S2B). Additionally, overexpression of LKB1 induced significant increases in the lipid levels and decreases in *bmm* expression in a dose-dependent manner (S3A and S3B Fig). The implication behind these observations is that LKB1 plays a critical role in lipid storage in *Drosophila* by regulating the lipolysis pathway in a kinase activity-dependent manner.

### SIK3 is a critical target of LKB1 for controlling lipid storage in *Drosophila*

To identify the lipolytic target of LKB1 among AMPK-related kinases in *Drosophila*, we determined mRNA levels of SIKs and AMPK, which are heavily involved in various metabolic pathways. As shown in Fig 1D, SIK3 and AMPKalpha were more highly expressed in the fat body. Furthermore, transgenic expression of constitutively active SIK3 (SIK3 T196E) in the fat body rescued the lipid accumulation and *bmm* expression defects of *LKB1*-null mutants, whereas expression of constitutively active AMPK (AMPK T184D) or inactive SIK3 with a mutation in the LKB1 phosphorylation site (SIK3 T196A) failed to rescue the lipid levels of the null mutants (Figs 1E, 1F, S4A and S4B). These results clearly suggest a specific role for SIK3 in the LKB1-mediated regulation of lipid storage in the fat body of *Drosophila*. Supporting this conclusion, overexpression of LKB1 highly augmented the phosphorylation of conserved Thr196 in SIK3 (Fig 1G), but this phosphorylation was completely lost in *LKB1* ^*X5*^ mutants (Fig 1H).

### *Drosophila SIK3* mutants display lipodystrophy

**Fig 2:**
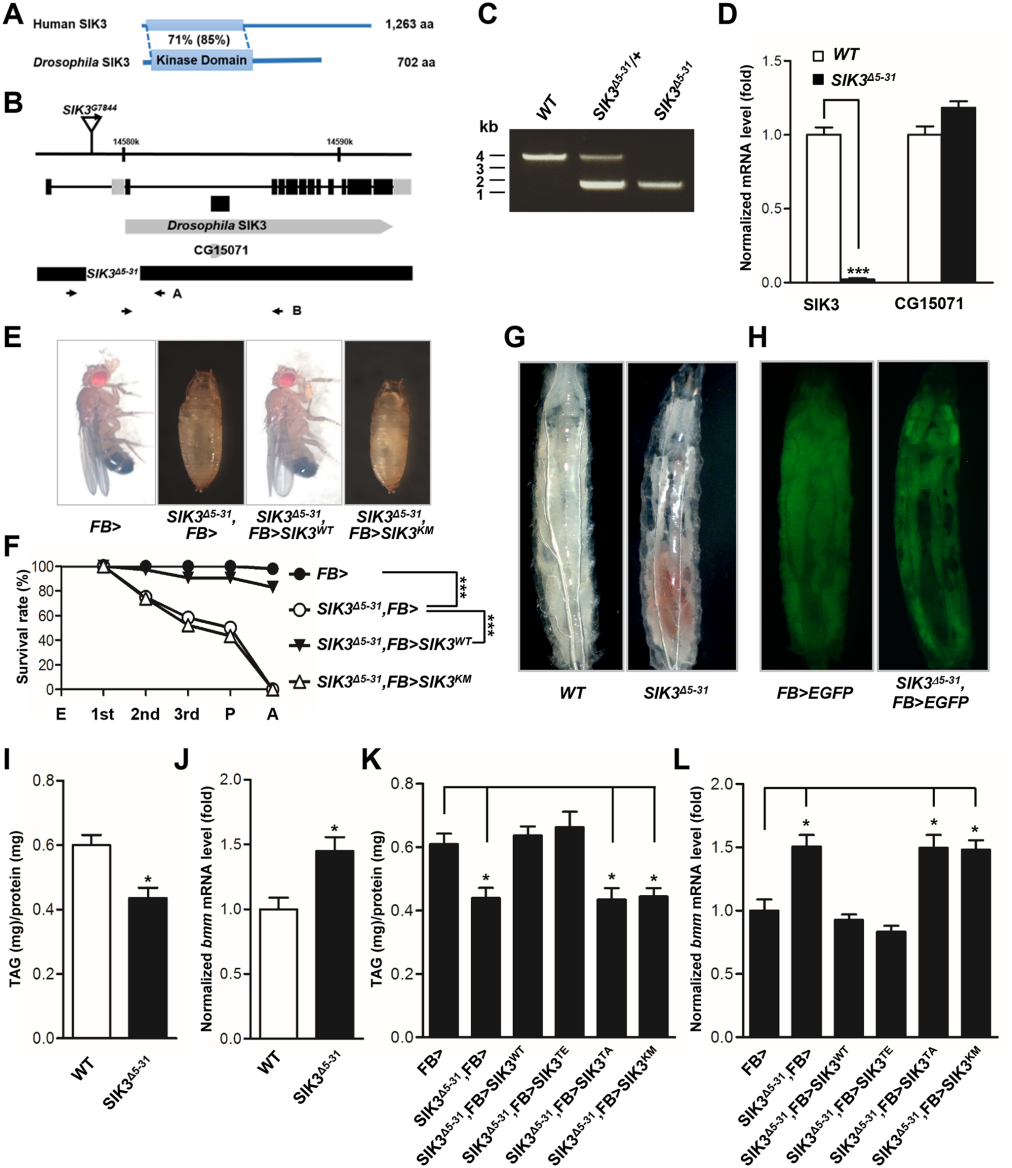
SIK3 null mutant is defective in lipid homeostasis and storage. (A) Amino acid sequence identities (similarities) of *Drosophila* SIK3 with human SIK3. (B) Genomic map of *Drosophila SIK3* locus. The exons of *SIK3* are indicated by boxes, and the coding regions are colored black. The deleted regions for *SIK3* null mutants (*SIK3* ^*Δ5–31*^) are also presented. (C) Genomic PCR analyses in wild-type (WT), heterozygous *SIK3* mutants (*SIK3* ^*Δ5–31*^/+) and *SIK3* ^*Δ5–31*^ using the A primer set in (B). (D) qPCR analysis for *SIK3*, using the B primer set in (B), and *CG15071* in wild-type and *SIK3* null mutant. (E) Restored viability of *SIK3* mutants by fat body-specific expression of wild-type SIK3, but not by kinase-dead (K70M) SIK3. (F) Relative survival rates in *SIK3* mutants with fat body-specific expression of wild-type and kinase-dead (K70M) SIK3 during development: embryo (E), first, second and third instar larva, pupa (P) and adult (A). Experimental and control survival rates are compared using the log-rank test (***P < 0.001). (E-F) Genotypes are as follows: FB> (*FB-Gal4*/+), SIK3^Δ5–31^,FB> (*FB-Gal4*,*SIK3* ^*Δ5–31*^ */SIK3* ^*Δ5–31*^), SIK3^Δ5–31^,FB>SIK3^WT^ (*FB-Gal4*,*SIK3* ^*Δ5–31*^ */SIK3* ^*Δ5–31*^ *;UAS-SIK3/+*), and SIK3^Δ5–31^,FB>SIK3^KM^ (*FB-Gal4*,*SIK3* ^*Δ5–31*^ */SIK3* ^*Δ5–31*^ *;UAS-SIK3 K70M/+*). (G) Wild-type and *SIK3* null mutant in the L3 larval stage. (H) EGFP expression in the fat body of wild-type and *SIK3* null mutant larvae. Genotypes are as follows: FB>EGFP (*FB-Gal4*/*UAS-2xEGFP*) and SIK3^Δ5–31^,FB>EGFP (*FB-Gal4*,*SIK3* ^*Δ5–31*^ */SIK3* ^*Δ5–31*^,*UAS-2xEGFP*). (I) TAG amounts of wild-type and *SIK3* null mutant larvae (*n* = 10 per genotype). (J) qPCR analysis of *bmm* in wild-type and *SIK3* null mutant larvae at mid-L2 stage under feeding condition. (K) TAG amounts in *SIK3* mutants with fat body-specific expression of wild-type, constitutively active (T196E), non-phosphorylable by LKB1 (T196A) or kinase-dead (K70M) SIK3. (*n* = 10 per genotype). (L) qPCR analysis of *bmm* in *SIK3* mutants following fat body-specific expression of wild-type, constitutively active (T196E) SIK3 or two kinase-dead SIK3 (T196A and K70M) at mid-L2 stage under feeding condition. (K-L) Genotypes are as follows: FB> (*FB-Gal4*/+), SIK3^Δ5–31^,FB> (*FB-Gal4*,*SIK3* ^*Δ5–31*^ */SIK3* ^*Δ5–31*^), SIK3^Δ5–31^,FB>SIK3^WT^ (*FB-Gal4*,*SIK3* ^*Δ5–31*^ */SIK3* ^*Δ5–31*^ *;UAS-SIK3/+*), SIK3^Δ5–31^,FB>SIK3^TE^ (*FB-Gal4*,*SIK3* ^*Δ5–31*^ */SIK3* ^*Δ5–31*^ *;UAS-SIK3 T196E/+*), SIK3^Δ5–31^,FB>SIK3^TA^ (*FB-Gal4*,*SIK3* ^*Δ531*^ */SIK3* ^*Δ5–31*^ *;UAS-SIK3 T196A/+*), and SIK3^Δ5–31^,FB>SIK3^KM^ (*FB-Gal4*,*SIK3* ^*Δ5–31*^ */SIK3* ^*Δ5–31*^ *;UAS-SIK3 K70M/+*). Data are presented as mean ± SEM (*P < 0.05; ***P < 0.001).

*Drosophila* SIK3, one of the AMPK-related kinases, shares considerable sequence homology with the kinase domain of mammalian SIK3 (Fig 2A). To assess the *in vivo* role of SIK3, *SIK3* loss-of-function mutants were generated by mobilizing the EP-element from *SIK3* ^*G7844*^ (Fig 2B). From 600 EP excision alleles, we generated *SIK3* ^*Δ5–31*^ mutant, which lacks 2,476 bp (2R14578001~14580477) that encodes for the translation start site and the ATP-binding site of SIK3 (Fig 2B and 2C). Confirming that *SIK3* ^*Δ5–31*^ is a null mutant, *SIK3* mRNA was not detected in the mutant (Fig 2D). However, the internal gene (CG15071) in the coding region of SIK3 was not affected (Fig 2D).

*SIK3* ^*Δ5–31*^ mutant flies died before the mid-pupal stage and showed a decreased survival rate (Fig 2E and 2F). The *SIK3* null mutant also exhibited a lipodystrophic phenotype (Fig 2G), and *FB-Gal4*-driven EGFP expression further confirmed the lean fat body phenotype of *SIK3* ^*Δ5–31*^ mutants compared to control flies (Fig 2H). Consistently, *SIK3* ^*Δ5–31*^ mutant had decreased lipid stores despite having a similar food intake in the larval stage (Figs 2I and S1A, respectively). Surprisingly, the fat body-specific expression of exogenous wild-type SIK3 rescued the lethality of *SIK3* ^*Δ5–31*^ mutant, while the expression of a kinase-dead SIK3 (SIK3 K70M) failed to rescue the mutant (Fig 2E and 2F). These results demonstrated that the phosphotransferase activity of SIK3 in the fat body is crucial for its function.

To further investigate the role of SIK3 in lipid metabolism, we analyzed *bmm* gene expression in *SIK3* ^*Δ5–31*^ mutant. Expectedly, the mutant showed markedly increased expression of *bmm* and increased lipase activity (Figs 2J and S1B, respectively), a phenotype similar to the LKB1 null mutant. Transgenic expression of either wild-type (SIK3 WT) or constitutively active SIK3 (SIK3 T196E) in the fat body of *SIK3* ^*Δ5–31*^ mutant resulted in full recovery of lipid levels and *bmm* expression compared to wild-type controls (Figs 2K, 2L, S2C and S2D). In contrast, expression of either inactive SIK3 harboring a mutation in the LKB1 phosphorylation site (SIK3 T196A) or a kinase-dead mutant (SIK3 K70M) failed to rescue the *SIK3* ^*Δ5–31*^ mutant phenotypes (Fig 2K and2L, respectively). Therefore, the kinase activity of SIK3 controlled by LKB1 is critical for the lipid storage in *Drosophila* fat body.

### The LKB1-SIK3 pathway is upstream of HDAC4, which regulates lipid storage and *bmm* expression

LKB1 and AMPK-related kinases play a major role in the inhibition of hepatic gluconeogenesis in response to high glucose levels via phosphorylation of the class IIa HDACs and the CREB co-activator CRTC [23–25]. To test whether HDAC4 or CRTC is involved in the LKB1 and SIK3 pathway, we analyzed the genetic interactions of LKB1 and SIK3 with HDAC4 and CRTC in *Drosophila*. We found that ablation of CRTC exacerbated the lethality of LKB1 and SIK3 null mutants (S5A and S5B Fig). However, strikingly, the loss of HDAC4 rescued the lethality of SIK3 null mutants, but did not affect the lethality of LKB1 null mutants (S6A–S6D Fig), suggesting that HDAC4 participates in LKB1-SIK3 signaling of *Drosophila*.

**Fig 3:**
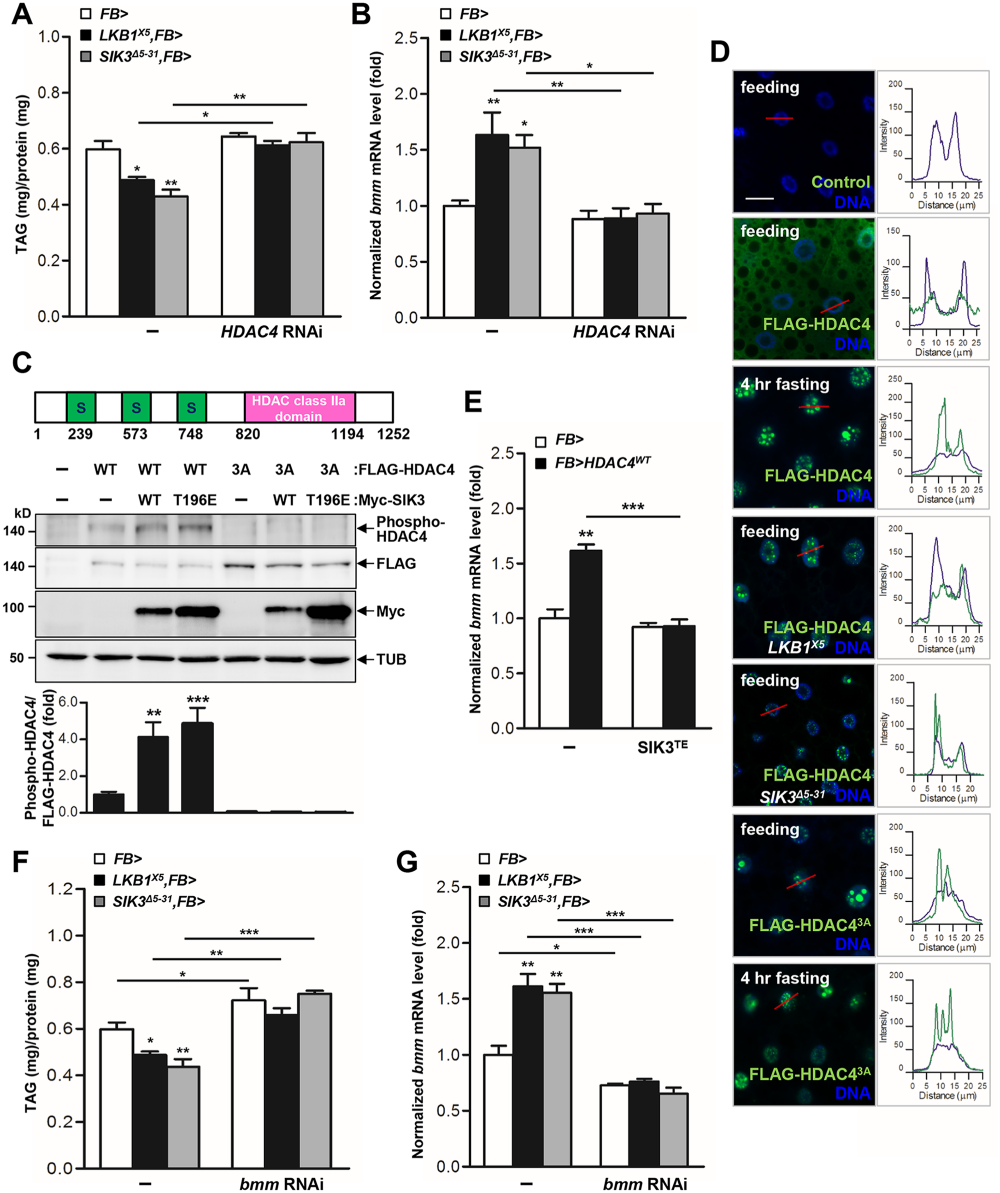
HDAC4 is the responsible target of LKB1-SIK3 signaling for controlling lipid homeostasis. (A-B) Effects of the fat body-specific knockdown of HDAC4 (*HDAC4* RNAi) on TAG amounts (A) and *bmm* gene expression (B) in *LKB1* and *SIK3* null mutants. Genotypes are as follows: FB> (*FB-Gal4*/+), LKB1^X5^,FB> (*FB-Gal4/+;LKB1* ^*X5*^ */LKB1* ^*X5*^), SIK3^Δ5–31^,FB> (*FB-Gal4*,*SIK3* ^*Δ5–31*^ */SIK3* ^*Δ5–31*^), FB>HDAC4 RNAi (*FB-Gal4/+;UAS-HDAC4 RNAi/+*), LKB1^X5^,FB>HDAC4 RNAi (*FB-Gal4/+;LKB1* ^*X5*^ */LKB1* ^*X5*^,*UAS-HDAC4 RNAi*), and SIK3^Δ5–31^,FB>HDAC4 RNAi (*FB-Gal4*,*SIK3* ^*Δ5–31*^ */SIK3* ^*Δ5–31*^ *;UAS-HDAC4 RNAi*). (C) *Drosophila* HDAC4 protein structure showing three SIK3 phosphorylation sites and an HDAC class IIa domain (top panel). Immunoblot analyses showing the effect of wild-type and constitutively active (T196E) SIK3 on HDAC4 Ser239 phosphorylation in larvae (middle four panels). Densitometric analyses of phospho-HDAC4 bands (bottom panel). *FB-Gal4* was used to drive transgene expression. Anti-phospho-Ser239 HDAC4, -FLAG (HDAC protein), -Myc (SIK3 protein), and -β-tubulin (TUB) antibodies were used. (D) Immunohistochemical analyses of HDAC4 (anti-FLAG antibody, green) in the fat body cells of wild type (first, second, and third rows), LKB1 mutant (*LKB1* ^*X5*^) (fourth row) and SIK3 mutant (*SIK3* ^*Δ5–31*^) (fifth row) L3 larvae in feeding or 4 hr fasting condition as denoted in figures. Similar experiments were also conducted for phosphorylation-defective and constitutively active HDAC4 (HDAC^3A^) in wild type L3 larvae in feeding (sixth row) or 4 hr fasting condition (bottom row). Cell nuclei were stained by Hoechst 33258 (blue). The graphs on the right of each image showed the intensity plot profile for each antibody staining along the red lines. Genotypes are as follows: Control (*FB-Gal4*/+), FLAG-HDAC4 (*FB-Gal4*/*UAS-HDAC4*), FLAG-HDAC4^3A^ (*FB-Gal4*/*UAS-HDAC4 3A*), FLAG-HDAC4,LKB1^X5^ (*FB-Gal4*/*UAS-HDAC4;LKB1* ^*X5*^/*LKB1* ^*X5*^), and FLAG-HDAC4,SIK3^Δ5–31^ (*FB-Gal4*,*SIK3* ^*Δ5–31*^/*UAS-HDAC4*,*SIK3* ^*Δ5–31*^). Scale bars, 20 μm. (E) Effect of fat body-specific expression of constitutively active SIK3 (SIK3 T196E) on *bmm* gene expression in larvae expressing wild-type HDAC4 in the fat body. Genotypes are as follows: FB> (*FB-Gal4*/+), FB>HDAC4^WT^ (*FB-Gal4/UAS-HDAC4*), FB>SIK3^TE^ (*FB-Gal4/+;UAS-SIK3 T196E/+*), and FB>HDAC4 ^WT^,SIK3^TE^ (*FB-Gal4/UAS-HDAC4;UAS-SIK3 T196E/+*). (F-G) Effects of the fat body-specific knockdown of bmm (*bmm* RNAi) on TAG amounts (F) and *bmm* gene expression (G) in *LKB1* and *SIK3* null mutants. Genotypes are as follows: FB> (*FB-Gal4*/+), LKB1^X5^,FB> (*FB-Gal4/+;LKB1* ^*X5*^ */LKB1* ^*X5*^), SIK3^Δ5–31^,FB> (*FB-Gal4*,*SIK3* ^*Δ5–31*^ */SIK3* ^*Δ5–31*^), FB>*bmm* RNAi (*FB-Gal4/;UAS-bmm RNAi/+*), LKB1^X5^,FB>*bmm* RNAi (*FB-Gal4/+;LKB1* ^*X5*^ */LKB1* ^*X5*^,*UAS-bmm RNAi*) and SIK3^Δ5–31^,FB>*bmm* RNAi (*FB-Gal4*,*SIK3* ^*Δ5–31*^ */SIK3* ^*Δ5–31*^ *;UAS-bmm RNAi*). Data are presented as mean ± SEM (*P < 0.05; **P < 0.01; ***P < 0.001).

To evaluate whether HDAC4 is crucial for the regulation of lipid storage by LKB1 and SIK3, we expressed HDAC4 RNAi in the fat body of LKB1 and SIK3 mutants. Surprisingly, knockdown of HDAC4 in the fat body fully rescued the TAG levels and *bmm* gene expression of LKB1 and SIK3 null mutants (Fig 3A and 3B, respectively), indicating that HDAC4 is indeed a critical downstream target of LKB1 and SIK3 in lipid metabolism of *Drosophila*.

### SIK3 induces the translocation of HDAC4 from the nucleus to the cytoplasm via phosphorylation

SIKs can regulate target gene expression by directly phosphorylating the class IIa HDACs and consequently inhibiting their translocation to the nucleus [26,27]. Expression of wild-type SIK3 (SIK3 WT) or constitutively active SIK3 (SIK3 T196E) augmented the phosphorylation of HDAC4 but not of the phosphorylation-defective HDAC4 (HDAC4 3A), demonstrating that SIK3 induces HDAC4 phosphorylation in *Drosophila* (Fig 3C). HDAC4 localized to both the cytoplasm and nuclei of larval fat body cells under feeding conditions, but localized mostly to the nucleus under fasting conditions (Fig 3D). However, HDAC4 accumulated in the nuclei of the fat body cells of LKB1 and SIK3 null mutants even under feeding conditions (Fig 3D). In addition, HDAC4 3A was retained in the nuclei of the fat body cells under both feeding and fasting conditions (Fig 3D). These results indicated that *Drosophila* SIK3, under the control of LKB1, phosphorylates HDAC4 in the fat body and regulates its nucleocytoplasmic localization in different dietary conditions.

The class IIa HDACs deacetylate and activate FOXO transcription factors [19,24], and the activated FOXO then induces ATGL/Bmm expression [19,28]. Overexpression of wild-type HDAC4 increased the mRNA levels of *bmm* (Fig 3E), indicating that HDAC4 regulates *bmm* gene expression in the fat body. Furthermore, overexpression of constitutively active SIK3 completely blocked the increased *bmm* expression induced by HDAC4 overexpression (Fig 3E), and *bmm* knockdown in the fat body blocked the decreases in TAG levels induced by LKB1 or SIK3 null mutation (Fig 3F and 3G). Altogether, these results suggested that the LKB1-SIK3 signaling pathway controls HDAC4-dependent Bmm activity in *Drosophila* fat body.

### LKB1-SIK3-HDAC4 signaling acts downstream of the AKH pathway to control lipid homeostasis

**Fig 4:**
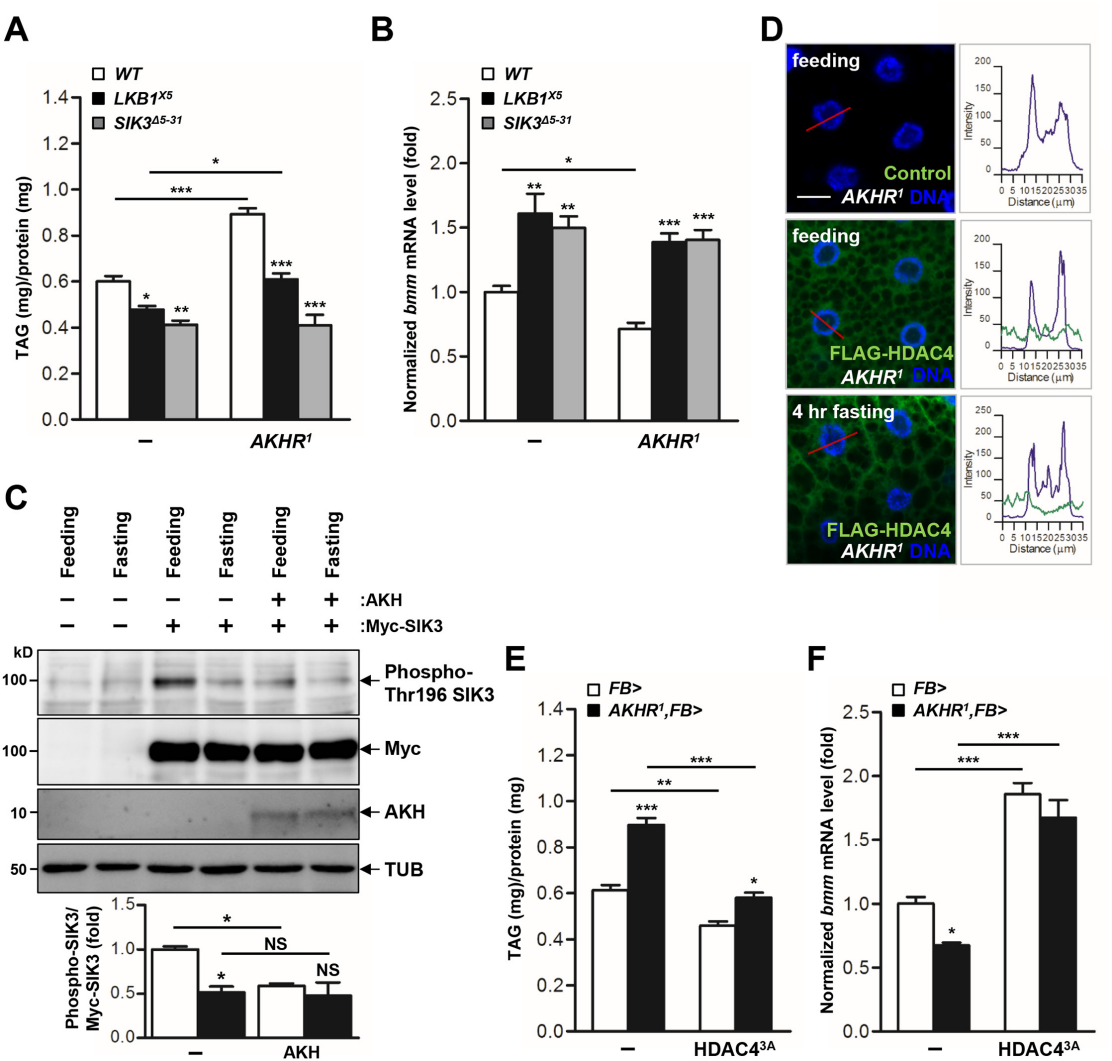
The AKH pathway regulates LKB1-SIK3-HDAC4 signaling to control lipid homeostasis. (A-B) Effects of AKHR gene disruption on TAG amounts (A) and *bmm* gene expression (B) in *LKB1* and *SIK3* null mutants. Genotypes are as follows: WT (*w* ^*1118*^), LKB1^X5^ (*LKB1* ^*X5*^/*LKB1* ^*X5*^), SIK3^Δ5–31^ (*SIK3* ^*Δ5–31*^ */SIK3* ^*Δ5–31*^), AKHR^1^ (*AKHR* ^*1*^ */AKHR* ^*1*^), LKB1^X5^,AKHR^1^ (*AKHR* ^*1*^ */AKHR* ^*1*^;*LKB1* ^*X5*^/*LKB1* ^*X5*^), and SIK3^Δ5–31^,AKHR^1^ (*SIK3* ^*Δ5–31*^,*AKHR* ^*1*^ */SIK3* ^*Δ5–31*^,*AKHR* ^*1*^). (C) Immunoblot analyses showing the effect of 4 hr fasting and AKH on Thr196 phosphorylation of SIK3 protein in larvae (top four panels). Densitometric analyses of phospho-SIK3 bands (bottom panel). *FB-Gal4* was used to drive transgene expression in the fat body. Anti-phospho-Thr196 SIK3, -Myc (SIK3 protein), -AKH and -β-tubulin (TUB) antibodies were used. (D) Immunohistochemical analyses of HDAC4 (anti-FLAG antibody, green) in AKHR mutant (*AKHR* ^*1*^) L3 larvae in feeding or 4 hr fasting condition as denoted in figures. Cell nuclei were stained by Hoechst 33258 (blue). Genotypes are as follows: Control (*FB-Gal4*,*AKHR* ^*1*^/*AKHR* ^*1*^) and FLAG-HDAC4,AKHR^1^ (*FB-Gal4*,*AKHR* ^*1*^/*UAS-HDAC4*,*AKHR* ^*1*^). The graphs showed the staining intensity profile for each antibody along the red lines. Scale bars, 20 μm. (E-F) Effect of the fat body-specific expression of constitutively active HDAC4 (HDAC^3A^) on TAG amounts (E) and *bmm* gene expression (F) in *AKHR* mutant. Genotypes: FB> (*FB-Gal4*/+), AKHR^1^,FB> (*FB-Gal4*,*AKHR* ^*1*^/*AKHR* ^*1*^), FB>HDAC4^3A^ (*FB-Gal4/UAS-HDAC4 3A*), and AKHR^1^,FB>HDAC4^3A^ (*FB-Gal4*,*AKHR* ^*1*^/*UAS-HDAC4 3A*,*AKHR* ^*1*^). Data are presented as mean ± SEM (*P < 0.05; **P < 0.01; ***P < 0.001; NS, non-significant).

Under fasting conditions, AKH activates the mobilization of fat body triglyceride by triggering AKHR and consequent activation of cAMP signaling in the fat body [7]. Consistently, we showed that AKHR mutation highly increased TAG levels (Fig 4A) and decreased *bmm* gene expression (Fig 4B). To determine the functional interaction between AKHR signaling and the LKB1-SIK3 signaling pathway, we crossed LKB1 or SIK3 null mutant flies with AKHR mutant flies. Interestingly, deletion of LKB1 or SIK3 reversed both the lipid accumulation and the reduced *bmm* expression phenotypes of AKHR mutant flies (Fig 4A and 4B, respectively), suggesting that the LKB1-SIK3 pathway likely acts downstream of AKHR. Furthermore, SIK3 Thr196 phosphorylation was reduced in both fasting and AKH overexpression conditions compared to that in feeding conditions (Fig 4C), supporting that the AKH pathway inhibits the kinase activity of LKB1 as shown by decreased SIK3 Thr196 phosphorylation.

On the basis of the observation that fasting induces the nuclear translocation of HDAC4, we also examined subcellular localization of HDAC4 in AKHR mutant flies. Intriguingly, HDAC4 in AKHR mutants localized to both the cytoplasm and the nucleus of larval fat body cells in 4 hr fasting condition compared to control (Figs 3D and 4D), suggesting that AKHR-dependent regulation is critical for HDAC4 localization. In addition, overexpression of the phosphorylation-defective HDAC4 by SIK3 (HDAC4 3A) suppressed the TAG levels and enhanced *bmm* expression in AKHR mutants (Fig 4E and 4F, respectively). Collectively, these results suggested that the LKB1-SIK3-HDAC4 pathway acts downstream of the AKH pathway to control lipolysis activity in *Drosophila*.

### AKH-independent signaling modulates the LKB1-SIK3-HDAC4 pathway under prolonged fasting

**Fig 5:**
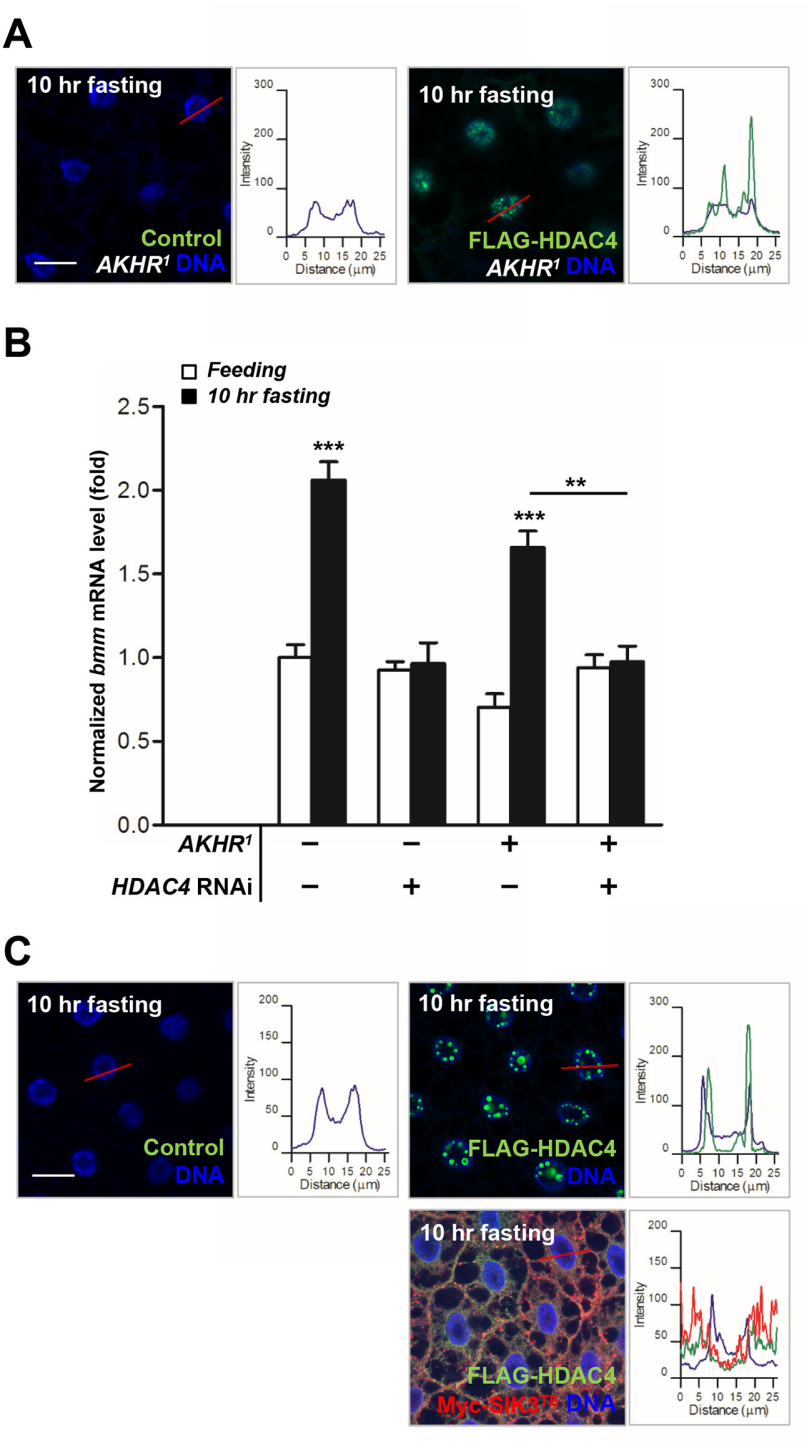
HDAC4 accumulated in the nuclei of the fat body cells in AKHR mutants under prolonged fasting. (A) Immunohistochemical analyses of HDAC4 (anti-FLAG antibody, green) in AKHR mutant (*AKHR* ^*1*^) L3 larvae in 10 hr fasting condition as denoted in figures. Cell nuclei were stained by Hoechst 33258 (blue). Genotypes are as follows: Control (*FB-Gal4*,*AKHR* ^*1*^/*AKHR* ^*1*^) and FLAG-HDAC4,AKHR^1^ (*FB-Gal4*,*AKHR* ^*1*^/*UAS-HDAC4*,*AKHR* ^*1*^). The graphs showed the staining intensity profile for each antibody along the red lines. Scale bars, 20 μm. (B) Effects of the fat body-specific knockdown of HDAC4 (*HDAC4* RNAi) on *bmm* gene expression in *AKHR* mutant adult flies (*AKHR* ^*1*^) in 10 hr fasting condition. Genotypes are as follows: Control (*FB-Gal4/+*), HDAC4 RNAi (*FB-Gal4/+;UAS-HDAC4 RNAi/+*), AKHR^1^ (*FB-Gal4*,*AKHR* ^*1*^/*AKHR* ^*1*^), and AKHR^1^,HDAC4 RNAi (*FB-Gal4*,*AKHR* ^*1*^/*AKHR* ^*1*^ *;UAS-HDAC4 RNAi/+*). (C) Immunohistochemical analyses of HDAC4 (anti-FLAG antibody, green) in the fat body cells following expression of constitutively active (T196E) SIK3 in 10 hr fasting condition as denoted in figures. Genotypes are as follows: Control (*FB-Gal4*/+), FLAG-HDAC4 (*FB-Gal4*/*UAS-HDAC4*), and FLAG-HDAC4,SIK3^TE^ (*FB-Gal4*/*UAS-HDAC4;UAS-SIK3 T196E/+*). The graphs showed the staining intensity profile for each antibody along the red lines. Scale bars, 20 μm. Data are presented as mean ± SEM (**P < 0.01; ***P < 0.001).

We showed that AKHR-dependent regulation of LKB1-SIK3 activity is critical for HDAC4 nuclear localization in ~4 hr fasting condition (Fig 4D). However, Gronke *et al*. showed that *bmm* gene expression is stimulated in AKHR mutant flies in 6 hr fasting condition [7]. Notably, in contrast to 4 hr fasting condition (Fig 4D), HDAC4 accumulated in the nuclei of the fat body cells in AKHR mutant flies after prolonged fasting (~10 hr) (Fig 5A), indicating that there should be AKHR-independent HDAC4 regulation during prolonged fasting. Furthermore, knockdown of HDAC4 in the fat body blocked the increased *bmm* gene expression in AKHR mutant flies after 10 hr fasting (Fig 5B), indicating that AKHR-independent signaling promotes HDAC4 nuclear localization to induce *bmm* gene expression under prolonged fasting conditions. Interestingly, expression of constitutively active SIK3 blocked the prolonged fasting-induced nuclear localization of HDAC4 (Fig 5C), suggesting that LKB1-SIK3 activity is critical for *bmm* expression under prolonged fasting. Taken together, these results demonstrated that the LKB1-SIK3-HDAC4 pathway acts as the primary lipolytic signaling upon both short-term and prolonged fasting while AKH plays a major role only in short-term fasting. Thus, it is of particular interest to investigate novel signaling mechanisms regulating the LKB1-SIK3-HDAC4 pathway under prolonged fasting conditions.

Our study provides evidence that LKB1 is necessary for maintaining *Drosophila* lipid storage via the regulation of lipolysis through the activation of SIK3. Consistent with our results in *Drosophila*, adipose tissue-specific LKB1 knockout mice showed decreased serum triglycerides [16], and the basal lipogenesis activity of adipocytes was significantly lower in LKB1 hypomorphic mice [29]. Recently, SIK3 null mice were also found to display a malnourished phenotype with lipodystrophy and were resistant to high-fat diets [17]. Thus, the LKB1-SIK3 pathway is indeed an evolutionally conserved regulatory mechanism for lipid homeostasis.

**Fig 6:**
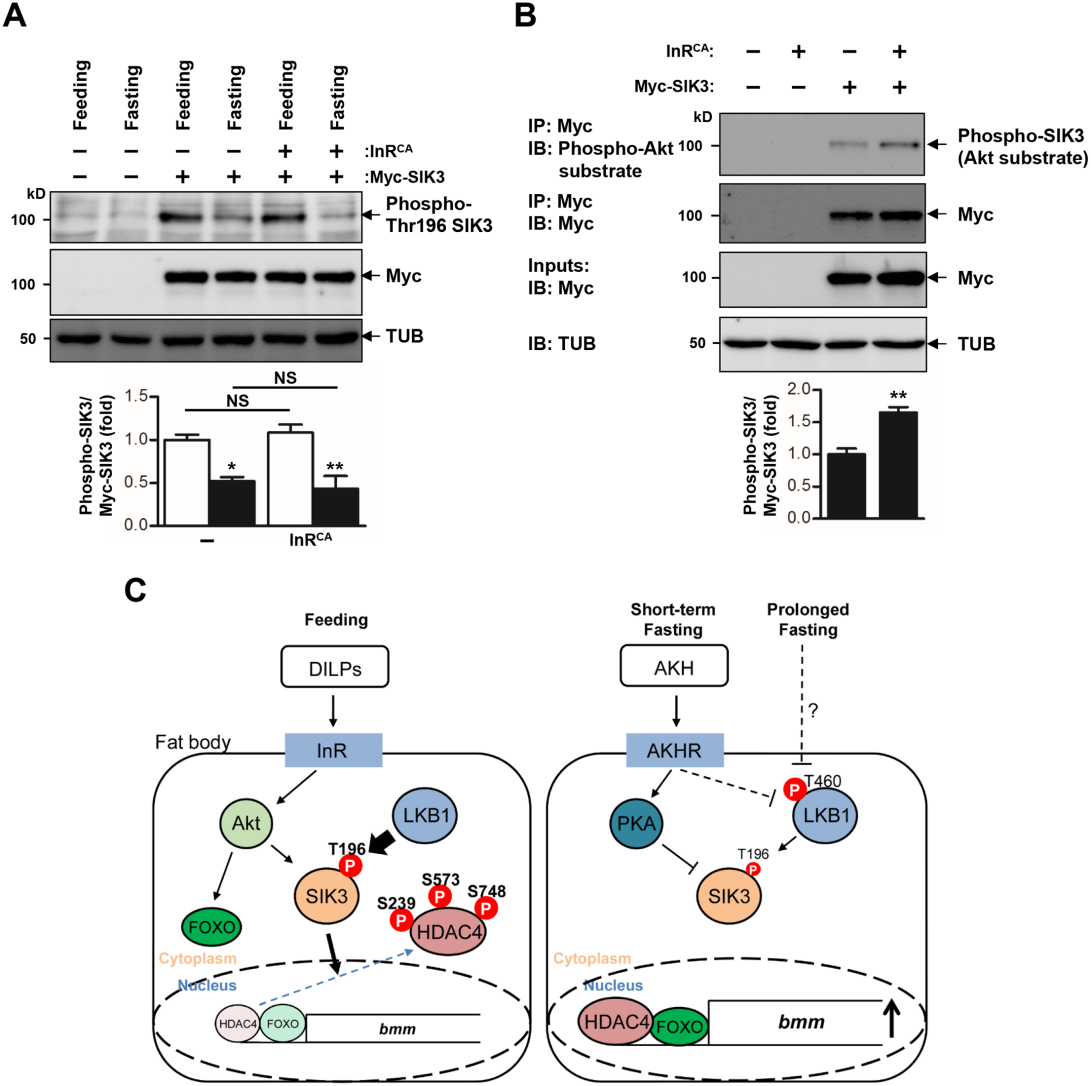
Activation of insulin receptor increases phosphorylation of SIK3 by Akt. (A) Immunoblot analyses showing the effect of 4 hr fasting and constitutively active insulin receptor (InR^CA^) on Thr196 phosphorylation of SIK3 protein in larvae (top three panels). Anti-phospho-Thr196 SIK3, -Myc (SIK3 protein), and -β-tubulin (TUB) antibodies were used. Densitometry of phospho-Thr196 SIK3 bands (bottom panel). *FB-Gal4* was used to drive transgene expression in the fat body. (B) Immunoblot analyses showing the effect of constitutively active insulin receptor (InR^CA^) on Akt-dependent phosphorylation of SIK3 protein in larvae (top four panels). The lysates were immunoprecipitated with an anti-Myc (SIK3 protein) antibody, and then immunoblotted with an anti-phospho-Akt substrate antibody. Densitometry of phospho-SIK3 bands (bottom panel). (C) A schematic model for LKB1 and SIK3 function to regulate lipid homeostasis in *Drosophila* fat body. LKB1 regulates the nucleocytoplasmic localization of HDAC4 via SIK3-dependent phosphorylation. Under feeding condition, DILPs-induced Akt activation leads to SIK3 activation, thereby inhibiting HDAC4 activity by phosphorylation. Under short-term fasting conditions, the AKH pathway inhibits the kinase activity of LKB1 in phosphorylating SIK3 Thr196 residue and controls SIK3 activity via PKA-dependent phosphorylation. Unphosphorylated and nuclear localized HDAC4 deacetylates and activates FOXO to increase *bmm* expression [19], thereby reducing lipid storage. AKH-independent signaling modulates the LKB1-SIK3-HDAC4 pathway to induce *bmm* expression when fasting is prolonged. Data are presented as mean ± SEM (*P < 0.05; **P < 0.01; NS, non-significant).

LKB1 is ubiquitously expressed and constitutively active in mammalian cells [15], which raises the question of how dietary conditions change the activity of LKB1 and SIK3 to control lipid homeostasis. Our findings suggested that fasting and the AKH pathway inhibit LKB1 activity to regulate SIK3 Thr196 phosphorylation (Figs 4C and 6C). It is possible that fasting- and AKH-induced inhibition of LKB1 activity can be achieved by altered subcellular localization, protein conformation, stability, and/or protein-protein interactions of LKB1 and its associated proteins. Interestingly, in HEK-293 cells, fasting triggers autophosphorylation of human LKB1 at Thr336 [30] that corresponds to Thr460 in *Drosophila* LKB1 [31]. This phosphorylation promotes the protein-protein interaction between LKB1 and 14-3-3 proteins [30] and inhibits the ability of LKB1 for suppressing cell growth [31].

In addition, the AKH pathway activates cAMP/PKA signaling in *Drosophila* [7]. Mammalian PKA inhibits SIK activity by phosphorylating a conserved serine residue [32,33] that corresponds to Ser563 in *Drosophila* SIK3 [19], suggesting that the AKH pathway also controls SIK3 activity via PKA-dependent phosphorylation (Fig 6C). On the other hand, the *Drosophila* insulin-like peptides (DILPs) did not increase SIK3 Thr196 phosphorylation (Fig 6A), but induced Akt-mediated SIK3 phosphorylation (Fig 6B), suggesting that DILPs directly regulate SIK3 activity independently of affecting LKB1 activity [19,34] (Fig 6C). Interestingly, these *Drosophila* signaling circuits are highly similar to mammalian insulin and glucagon pathways in controlling lipid metabolism and storage, raising questions regarding whether the LKB1-SIK3-HDAC4 signaling pathway is also conserved in mammalian systems as a converging point between feeding and fasting signals to control lipid homeostasis.

Is SIK3 also involved in the modulation of other LKB1 functions, such as the regulation of cell polarity and mitosis? SIK3 null mutants showed normal epithelial polarity and mitosis (S7A and S7B Fig). Additionally, transgenic expression of constitutively active SIK3 (SIK3 T196E) failed to suppress the cell polarity and mitosis defects of LKB1 mutants (S8A and S8B Fig), suggesting that SIK3 does not participate in the regulation of cell polarity and mitosis by LKB1. In addition, both fat body-specific expression of LKB1 and ablation of HDAC4 failed to rescue the lethality of LKB1 null mutants (S6C Fig), indicating that LKB1 has SIK3/HDAC4-independent roles and additional targets in other tissues and developmental processes.

In summary, we have demonstrated that the LKB1-SIK3 pathway is important for maintaining lipid homeostasis in *Drosophila*. As alterations in lipolysis are closely associated with human obesity [35], future studies will be required to unravel the relationship between LKB1-SIK3-HDAC4 signaling and obesity-related metabolic diseases.

## Materials and Methods

### Fly strains

The following fly stocks were used in this study: *LKB1* ^*X5*^, *UAS-LKB1* ^*WT*^, and *UAS-LKB1* ^*KI*^ [22], *HDAC4* ^*KG09091*^ (Bloomington #15159), *UAS-HDAC4 RNAi* (VDRC #20522), *UAS-bmm RNAi* (Bloomington #25926), *UAS-InR* ^*CA*^ (Bloomington #15159), *cg-Gal4* (Bloomington #7011), *hs-Gal4* (Bloomington #1799), *UAS-2xEGFP* (Bloomington #6874), *UAS-HA-AMPK* ^*TD*^ [12], *CRTC* ^*25-3*^ [36], *UAS-FLAG-HDAC4* ^*WT*^ and *UAS-FLAG-HDAC4* ^*3A*^ [19], *AKHR* ^*1*^ [7], and *FB-Gal4* [37]. *SIK3* ^*Δ5–31*^ was generated by imprecise excision of *SIK3* ^*G7844*^ line (KAIST *Drosophila* Library Facility, Daejeon, Korea). To generate *UAS-SIK3* flies, *SIK3* EST cDNA (Berkeley *Drosophila* Genome Project accession no. LD07105) was cloned into the Myc-tagged pUAST vector and microinjected into *w* ^*1118*^ embryos. All flies were grown on food containing approximately 35 g cornmeal, 70 g dextrose, 5 g agar, 50 g dry active yeast (Ottogi, Inc., Korea), 4.6 ml propionic acid, and 7.3 ml Tegosept (100 g/l in ethanol) per liter at 25°C. All flies were backcrossed for a minimum of 6 generations into *w* ^*1118*^ background.

### Site-directed mutagenesis

The QuickChange kit (Stratagene) was used for site-directed mutagenesis. For generation of a kinase-dead mutant SIK3 (Lys70Met, SIK3^K70M^), 5’-CAAGACAAAGGTGGCCATCATGATCATAGACAAAACATGTC-3’ and 5’-GACATGTTTTGTCTATGATCATGATGGCCACCTTTGTCTTG-3’ primers were used. For generation of a SIK3 mutant non-phosphorylatable by LKB1 (Thr196Ala, SIK3 T196A), 5’-GGGTGCCACCTTAAAAGCTTGGTGTGGATCAC-3’ and 5’-GTGATCCACACCAAGCTTTTAAGGTGGCACCC-3’ primers were used. For generation of a SIK3 mutant mimicking LKB1-dependent phosphorylation (Thr196Glu, SIK3 T196E), 5’-GAGGGTGCCACCTTAAAAGAATGGTGTGGATCACCGCCC-3’ and 5’-GGGCGGTGATCCACACCATTCTTTTAAGGTGGCACCCTC-3’ primers were used.

### qPCR analysis

Larvae were collected, and RNA was extracted using the RNeasy Mini Kit (QIAGEN). Total RNA (1 μg) was reverse-transcribed by M-MLV Reverse Transcriptase (Promega) to generate cDNA for quantitative real-time RT-PCR (Bio-Rad CFX96 Real-Time PCR detection system, SYBR Green) using a 500 nM primer concentration and 2 ng of cDNA template. The primers were used in S1 Table. The relative values were calculated using the ΔΔCt method via normalization to *rp49* mRNA levels. Results were expressed in arbitrary units, with each control value as 1 unit.

### Immunohistochemistry

Third instar larvae were dissected in *Drosophila* Ringer’s solution and fixed with 4% formaldehyde in phosphate buffered saline (PBS) for 10 min at room temperature. After being washed with 0.1% Triton X-100 in PBS (PBST), the samples were blocked for 1 hr incubation at room temperature with 5% bovine serum albumin (BSA) in PBST. The samples were further incubated at 4°C for 16 hr with the indicated antibodies: anti-FLAG-M2 (Sigma, F1804), anti-Myc (Cell Signaling Technology, #2272), anti-aPKC (Santa Cruz, sc-216), and anti-PH3 (Millipore, 06–570). Following three washes with PBST, the samples were incubated with appropriate secondary antibodies (and with Hoechst 33258 used for staining DNA, if required) for 3 hr at room temperature. The samples were washed with PBST and mounted with 80% glycerol in PBS, then observed by a confocal microscope LSM710 (Zeiss).

### Feeding assay

Feeding assay was performed according to previously described with minor modifications [38]. Blue food dye (Erioglaucine Disodium Salt, Sigma, #861146) was added at 1% (w/v) to fly food. Larvae were switched from normal food to blue-color food for 2 hr. After feeding, larvae were frozen immediately. Samples were homogenized in PBS buffer and centrifuged for 25 min at 13,200 rpm. The absorbance of the supernatant was measured at 625 nm using Infinite M200 spectrophotometer (Tecan).

### Immunoblot analysis

Larvae were lysed in a lysis buffer (20 mM Tris-HCl (pH 7.5), 1 mM EDTA, 5 mM EGTA, 150 mM NaCl, 20 mM NaF, 1% Triton X-100, 1 μg/ml leupeptin, and 1mM PMSF) for 30–60 min on ice. After centrifugation for 15 min at 13,200 rpm, supernatants were reserved for SDS-PAGE analysis, and proteins were then transferred to nitrocellulose membranes (GE Healthcare, #BA85). Membranes were incubated in a blocking solution (Tris-buffered saline (TBS) containing 0.1% Tween-20, 5% BSA) for 1hr. The primary antibodies used were anti-LKB1 [22], anti-phospho-Thr196 SIK3 [39], anti-phospho-Ser239 HDAC4 (Cell Signaling Technology, #3443), anti-phospho-Akt substrate (Cell Signaling Technology, #9614), anti-FLAG-M2 (Sigma, #F1804), anti-Myc (Cell Signaling Technology, #2272), anti-AKH (a gift from Dr. Veenstra), and anti-β-tubulin antibody (Developmental Studies Hybridoma Bank, E7). Protein detection was done using the LAS-4000 imaging system (Fujifilm), and densitometric analysis was performed using Multi Gauge 3.0 software.

### Lipid measurement and lipase assay

TAG measurement was performed according to previously described methods using Free Glycerol Reagent (Sigma, #F6428) and Triglyceride Reagent (Sigma, #T2449) [40]. A standard curve was generated with a glycerol standard solution (Sigma, #G7793). Samples were assayed at 540 nm using Infinite M200 spectrophotometer (Tecan). In each homogenate, amounts of TAG (in mg) were normalized to those of protein (in mg) using Bradford protein assay (Bio-Rad). Lipase activity was determined according to the manufacturer’s instructions with QuantiChrom kit (BioAssay Systems, DLPS-100).

### Survival rate analysis

The analysis for survival rate was performed as previously described [12]. The eggs from flies with the appropriate genotypes were laid on 60 mm dishes containing standard apple juice-agar with yeast paste for 4 hr. The hatched larvae were collected using selection markers and transferred to plates containing normal food media. The green balancer chromosome (*CyO*, *Actin-GFP*) was used to select the homozygous *SIK3* ^*Δ5–31*^ mutant. The number of larvae was scored for viability at each developmental stage, and dead larvae were removed. At least 100 larvae were studied per genotype.

### Statistical analysis

All quantitative data are analyzed using Student’s *t* tests or ANOVA with a post Tukey’s multiple comparison test, and *P* < 0.05 was considered statistically significant. Each experiment was repeated at least three times, data are presented as the average ± standard error of the mean (SEM). The *P* values given in the survival data are the result of a log rank test using GraphPad Prism 5 software.

## Supporting Information

S1 Fig LKB1 and SIK3 null mutants showed elevated lipase activity with similar food intakes. (A) Blue dye feeding assay in LKB1 mutant (LKB1^X5^) and SIK3 mutant (SIK3^Δ5–31^) at L2 (48 hr AEL) stage, normalized to wild-type larvae. Absorbance of the blue dye was measured at 625 nm. (B) Lipase activity (1 unit is defined as the cleavage of 1μmol substrate per minute) were determined in wild type, LKB1 mutant (LKB1^X5^), and SIK3 mutant (SIK3^Δ5–31^) at L2 (48 hr AEL) stage. (A-B) Genotypes are as follows: WT (*w* ^*1118*^), LKB1^X5^ (*LKB1* ^*X5*^/*LKB1* ^*X5*^), and SIK3^Δ5–31^ (*SIK3* ^*Δ5–31*^ */SIK3* ^*Δ5–31*^). Data are presented as mean ± SEM (*P < 0.05; NS, non-significant).
(TIF)

S2 Fig Transgenic expression with *cg-Gal4*, a fat body driver, also restored the lipid storage of LKB1 and SIK3 mutants. (A-B) TAG amounts (A) and qPCR analysis of *bmm* mRNA amounts (B) in *LKB1* mutants following fat body-specific expression of wild-type LKB1. Genotypes are as follows: cg> (*cg-Gal4*/+), LKB1^X5^,cg> (*cg-Gal4/+;LKB1* ^*X5*^ */LKB1* ^*X5*^), and LKB1^X5^,cg>LKB1^WT^ (*cg-Gal4/UAS-LKB1;LKB1* ^*X5*^ */LKB1* ^*X5*^). (C-D) TAG amounts (C) and qPCR analysis of *bmm* mRNA amounts (D) in *SIK3* mutants following fat body-specific expression of wild-type SIK3. Genotypes are as follows: cg> (cg-Gal4/+), SIK3^Δ5–31^,cg> (*cg-Gal4/SIK3* ^*Δ5–31*^ */SIK3* ^*Δ5–31*^), and SIK3^*Δ*5–31^,cg>SIK3^WT^ (*cg-Gal4*,*SIK3* ^*Δ5–31*^ */SIK3* ^*Δ5–31*^ *;UAS-SIK3/+*). Data are presented as mean ± SEM (*P < 0.05).
(TIF)

S3 Fig LKB1 increased lipid storage in a gene dosage-dependent manner. (A-B) Effects of fat body-specific expression of one copy and two copies of wild-type LKB1 on TAG amounts (A) and qPCR analysis of *bmm* mRNA amounts (B) in wild-type larvae. Genotypes are as follows: FB> (*FB-Gal4*/+), FB>LKB1 (*FB-Gal4/+;UAS-LKB1/+*), and FB>LKB1 (2X) (*FB-Gal4/+;UAS-LKB1/UAS-LKB1*). Data are presented as mean ± SEM (*P < 0.05; **P < 0.01; NS, non-significant).
(TIF)

S4 Fig Transgenic expression of an inactive SIK3 with a mutation in LKB1 phosphorylation site (SIK3 T196A) failed to rescue the lipid storage phenotype of LKB1 mutants. (A-B) TAG amounts (A) and qPCR analysis of *bmm* mRNA amounts (B) in *LKB1* mutants following fat body-specific expression of a SIK3 mutant non-phosphorylable by LKB1 (SIK3 T196A). Genotypes are as follows: FB> (*FB-Gal4*/+), LKB1^X5^,FB> (*FB-Gal4/+;LKB1* ^*X5*^ */LKB1* ^*X5*^), FB>SIK3^TA^ (*FB-Gal4/UAS-SIK3 T196A*), and LKB1^X5^,FB>SIK3^TA^ (*FB-Gal4/UAS-SIK3 T196A;LKB1* ^*X5*^ */LKB1* ^*X5*^). Data are presented as mean ± SEM (*P < 0.05; **P < 0.01).
(TIF)

S5 Fig Loss of CRTC failed to rescue the lethality of LKB1 and SIK3 null mutants. (A) Effect of CRTC gene disruption on fly development in *LKB1* and *SIK3* mutants. (B) Relative survival rates in *LKB1* and *SIK3* mutants with CRTC gene disruption during development: embryo (E), first, second and third instar larva, pupa (P) and adult (A). Experimental and control survival rates are compared using the log-rank test (**P < 0.01; ***P < 0.001; NS, non-significant). (A-B) Genotypes are as follows: WT (*w* ^*1118*^), LKB1^X5^ (*LKB1* ^*X5*^/*LKB1* ^*X5*^), SIK3^Δ5–31^ (*SIK3* ^*Δ5–31*^ */SIK3* ^*Δ5–31*^), CRTC^25-3^ (*CRTC* ^*25-3*^ */CRTC* ^*25-3*^), LKB1^X5^,CRTC^25-3^ (*LKB1* ^*X5*^,*CRTC* ^*25-3*^ */LKB1* ^*X5*^,*CRTC* ^*25-3*^), and SIK3^Δ5–31^,CRTC^25-3^ (*SIK3* ^*Δ5–31*^ */SIK3* ^*Δ5–31*^ *;CRTC* ^*25-3*^ */CRTC* ^*25-3*^).
(TIF)

S6 Fig Loss of HDAC4 rescued the lethality of SIK3 mutants. (A) Genomic region of *HDAC4* locus. Exons of *HDAC4* are indicated by boxes, and coding regions are colored black. The region for *HDAC4* hypomorphic mutants (*HDAC4* ^*KG09091*^) is presented. (B) qPCR analysis of *HDAC4* mRNA levels in wild-type and *HDAC4* mutant adult flies. Data are presented as mean ± SEM (**P < 0.01). (C-D) Effects of HDAC4 gene disruption on fly development (C) and survival rates (D) in *LKB1* and *SIK3* mutants. Experimental and control survival rates are compared using the log-rank test (***P < 0.001; NS, non-significant). Genotypes are as follows: WT (*w* ^*1118*^), LKB1^X5^ (*LKB1* ^*X5*^/*LKB1* ^*X5*^), SIK3^Δ5–31^ (*SIK3* ^*Δ5–31*^ */SIK3* ^*Δ5–31*^), HDAC4^KG09091^ (*HDAC4* ^*KG09091*^), LKB1^X5^,HDAC4^KG09091^ (*HDAC4* ^*KG09091*^ *;;LKB1* ^*X5*^/*LKB1* ^*X5*^), and SIK3^Δ5–31^,HDAC4^KG09091^ (*HDAC4* ^*KG09091*^ *;SIK3* ^*Δ5–31*^ */SIK3* ^*Δ5–31*^).
(TIF)

S7 Fig SIK3 mutants show normal epithelial polarity and mitosis. Wing discs (A) and brain hemispheres (B) in wild-type and SIK3 null mutant larvae stained with anti-aPKC (apical complex marker, green) antibody and Hoechst 33258 (DNA, blue) (A), or with anti-PH3 antibody (B). Vertical images were obtained from the Z-stack. Scale bars, white, 10 mm; yellow, 50 mm. Genotypes are as follows: WT (*w* ^*1118*^) and SIK3^Δ5–31^ (*SIK3* ^*Δ5–31*^ */SIK3* ^*Δ5–31*^).
(TIF)

S8 Fig The epithelial polarity and mitosis defects in LKB1 mutants were not restored by constitutively active SIK3 expression. Wing discs (A) and brain hemispheres (B) in wild-type, LKB1 null mutant, and LKB1 null expressing SIK3^TE^ (LKB1^X5^, hs (heat shock)>SIK3 TE) larvae stained with anti-aPKC (apical complex marker, green) antibody and Hoechst 33258 (DNA, blue) (A), or with anti-PH3 antibody (B). Vertical images were obtained from the Z-stack. The white arrows indicate mitotic chromosomes with polyploidy. Scale bars, white, 10 mm; yellow, 50 mm. Genotypes are as follows: hs> (*hs-Gal4/+*), LKB1^X5^,hs> (*hs-Gal4/+;LKB1* ^*X5*^/*LKB1* ^*X5*^), and LKB1^X5^,hs>SIK3^TE^ (*hs-Gal4/UAS-SIK3 T196E;LKB1* ^*X5*^/*LKB1* ^*X5*^).
(TIF)

S1 Table List of qPCR primers used in this study. (DOCX)
