# Supplementary figures and images for: Feeding and Fasting Signals Converge on the LKB1-SIK3 Pathway to Regulate Lipid Metabolism in Drosophila
Source: PLoS Genet. 2015 May 21;11(5):e1005263. doi: 10.1371/journal.pgen.1005263 (PMC4440640; doi:10.1371/journal.pgen.1005263)

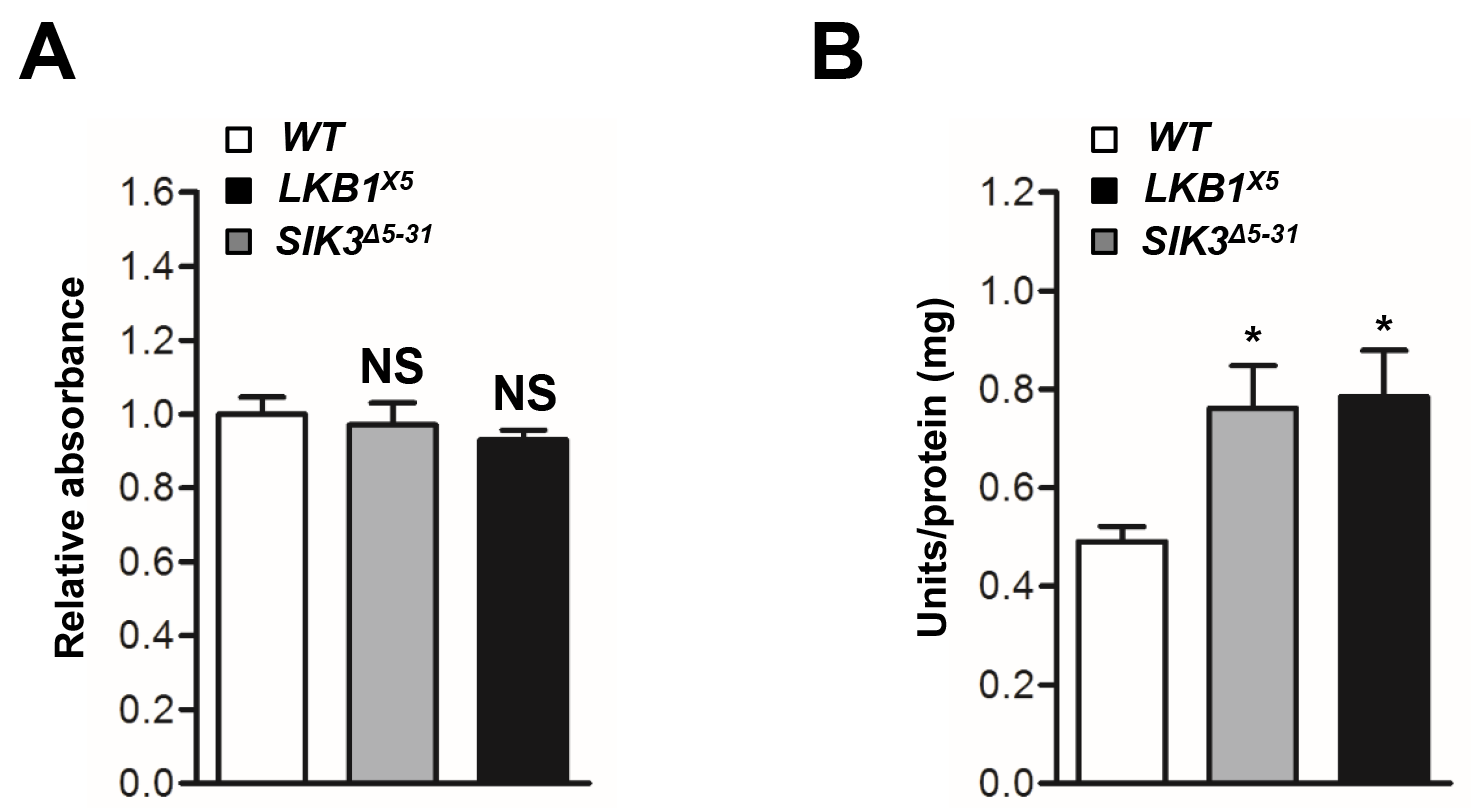

Supplement: S1 Fig — (A) Blue dye feeding assay in LKB1 mutant (LKB1X5) and SIK3 mutant (SIK3Δ5–31) at L2 (48 hr AEL) stage, normalized to wild-type larvae. Absorbance of the blue dye was measured at 625 nm. (B) Lipase activity (1 unit is defined as the cleavage of 1μmol substrate per minute) were determined in wild type, LKB1 mutant (LKB1X5), and SIK3 mutant (SIK3Δ5–31) at L2 (48 hr AEL) stage. (A-B) Genotypes are as follows: WT (w 1118), LKB1X5 (LKB1 X5/LKB1 X5), and SIK3Δ5–31 (SIK3 Δ5–31 /SIK3 Δ5–31). Data are presented as mean ± SEM (*P < 0.05; NS, non-significant). (TIF) [file pgen.1005263.s001.tif]

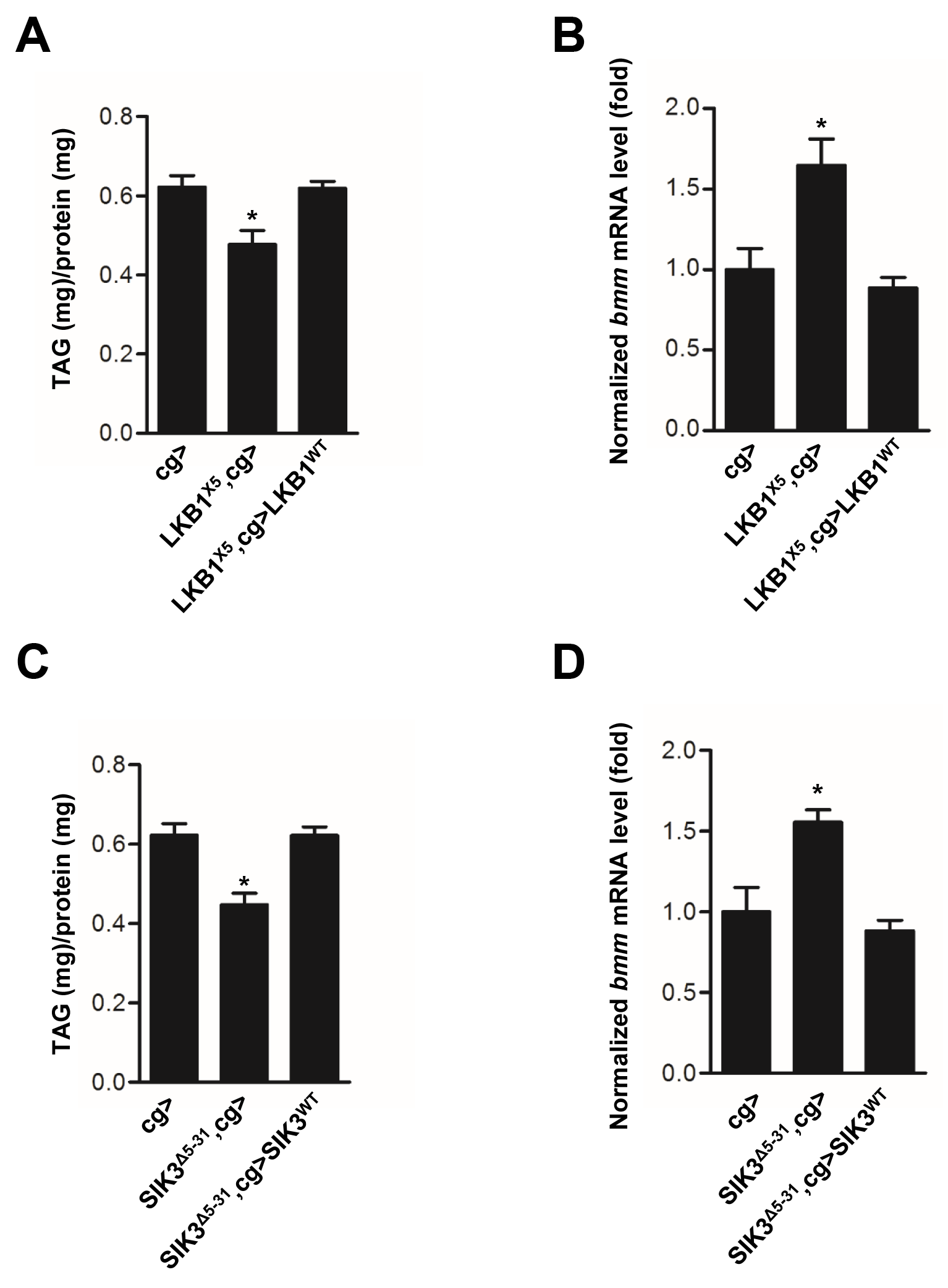

Supplement: S2 Fig — (A-B) TAG amounts (A) and qPCR analysis of bmm mRNA amounts (B) in LKB1 mutants following fat body-specific expression of wild-type LKB1. Genotypes are as follows: cg> (cg-Gal4/+), LKB1X5,cg> (cg-Gal4/+;LKB1 X5 /LKB1 X5), and LKB1X5,cg>LKB1WT (cg-Gal4/UAS-LKB1;LKB1 X5 /LKB1 X5). (C-D) TAG amounts (C) and qPCR analysis of bmm mRNA amounts (D) in SIK3 mutants following fat body-specific expression of wild-type SIK3. Genotypes are as follows: cg> (cg-Gal4/+), SIK3Δ5–31,cg> (cg-Gal4/SIK3 Δ5–31 /SIK3 Δ5–31), and SIK3Δ5–31,cg>SIK3WT (cg-Gal4,SIK3 Δ5–31 /SIK3 Δ5–31 ;UAS-SIK3/+). Data are presented as mean ± SEM (*P < 0.05). (TIF) [file pgen.1005263.s002.tif]

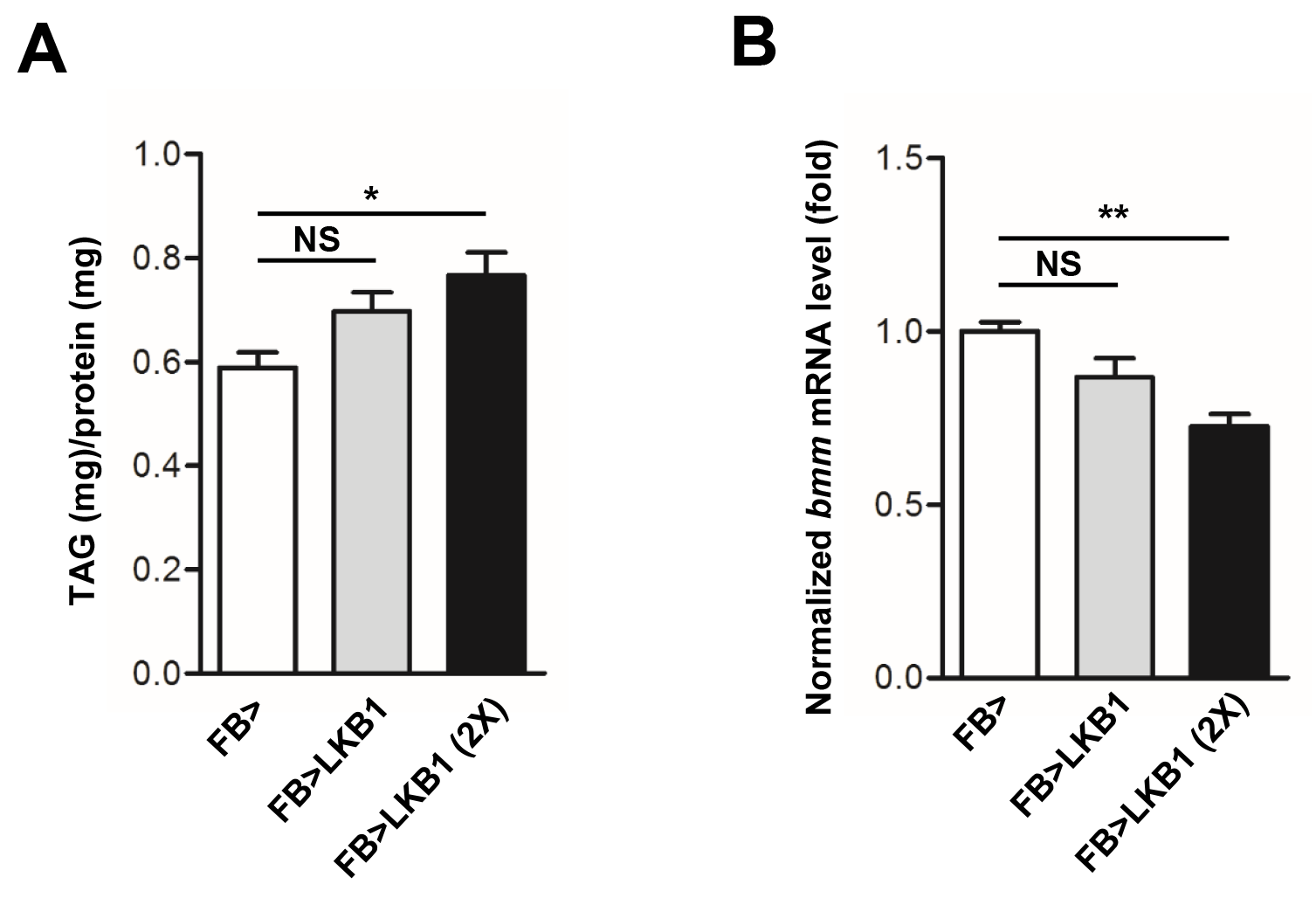

Supplement: S3 Fig — (A-B) Effects of fat body-specific expression of one copy and two copies of wild-type LKB1 on TAG amounts (A) and qPCR analysis of bmm mRNA amounts (B) in wild-type larvae. Genotypes are as follows: FB> (FB-Gal4/+), FB>LKB1 (FB-Gal4/+;UAS-LKB1/+), and FB>LKB1 (2X) (FB-Gal4/+;UAS-LKB1/UAS-LKB1). Data are presented as mean ± SEM (*P < 0.05; **P < 0.01; NS, non-significant). (TIF) [file pgen.1005263.s003.tif]

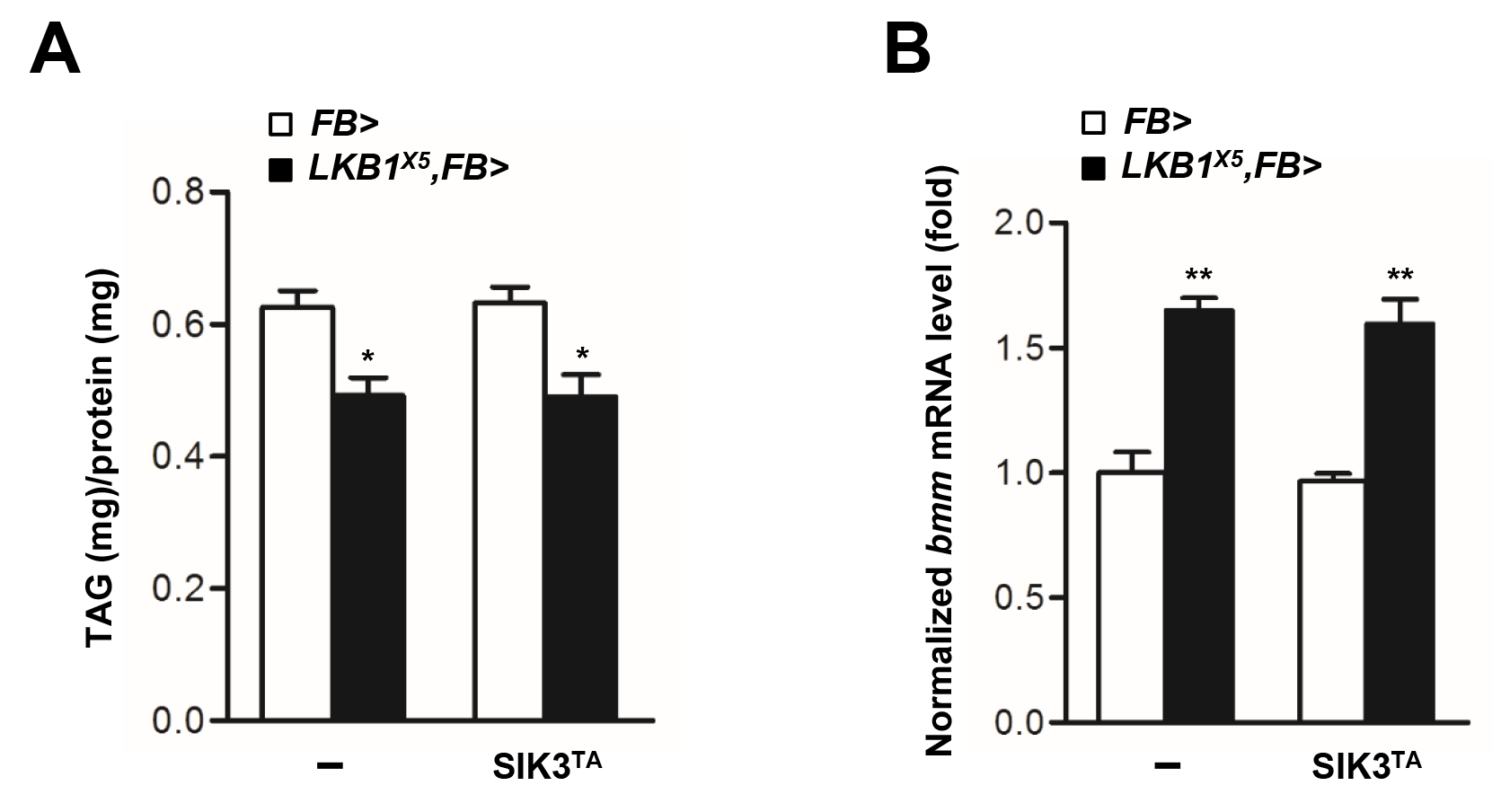

Supplement: S4 Fig — (A-B) TAG amounts (A) and qPCR analysis of bmm mRNA amounts (B) in LKB1 mutants following fat body-specific expression of a SIK3 mutant non-phosphorylable by LKB1 (SIK3 T196A). Genotypes are as follows: FB> (FB-Gal4/+), LKB1X5,FB> (FB-Gal4/+;LKB1 X5 /LKB1 X5), FB>SIK3TA (FB-Gal4/UAS-SIK3 T196A), and LKB1X5,FB>SIK3TA (FB-Gal4/UAS-SIK3 T196A;LKB1 X5 /LKB1 X5). Data are presented as mean ± SEM (*P < 0.05; **P < 0.01). (TIF) [file pgen.1005263.s004.tif]

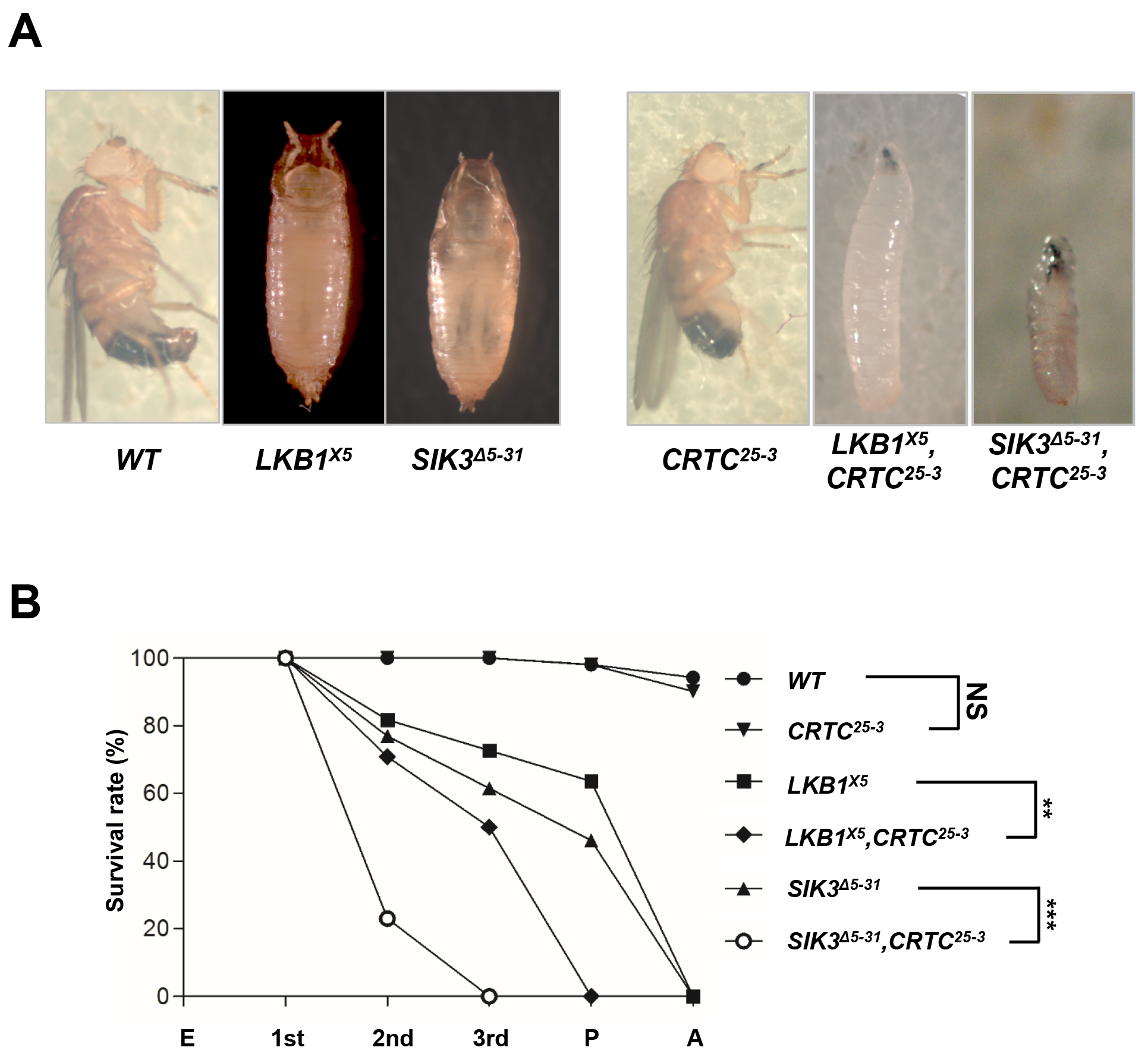

Supplement: S5 Fig — (A) Effect of CRTC gene disruption on fly development in LKB1 and SIK3 mutants. (B) Relative survival rates in LKB1 and SIK3 mutants with CRTC gene disruption during development: embryo (E), first, second and third instar larva, pupa (P) and adult (A). Experimental and control survival rates are compared using the log-rank test (**P < 0.01; ***P < 0.001; NS, non-significant). (A-B) Genotypes are as follows: WT (w 1118), LKB1X5 (LKB1 X5/LKB1 X5), SIK3Δ5–31 (SIK3 Δ5–31 /SIK3 Δ5–31), CRTC25-3 (CRTC 25-3 /CRTC 25-3), LKB1X5,CRTC25-3 (LKB1 X5,CRTC 25-3 /LKB1 X5,CRTC 25-3), and SIK3Δ5–31,CRTC25-3 (SIK3 Δ5–31 /SIK3 Δ5–31 ;CRTC 25-3 /CRTC 25-3). (TIF) [file pgen.1005263.s005.tif]

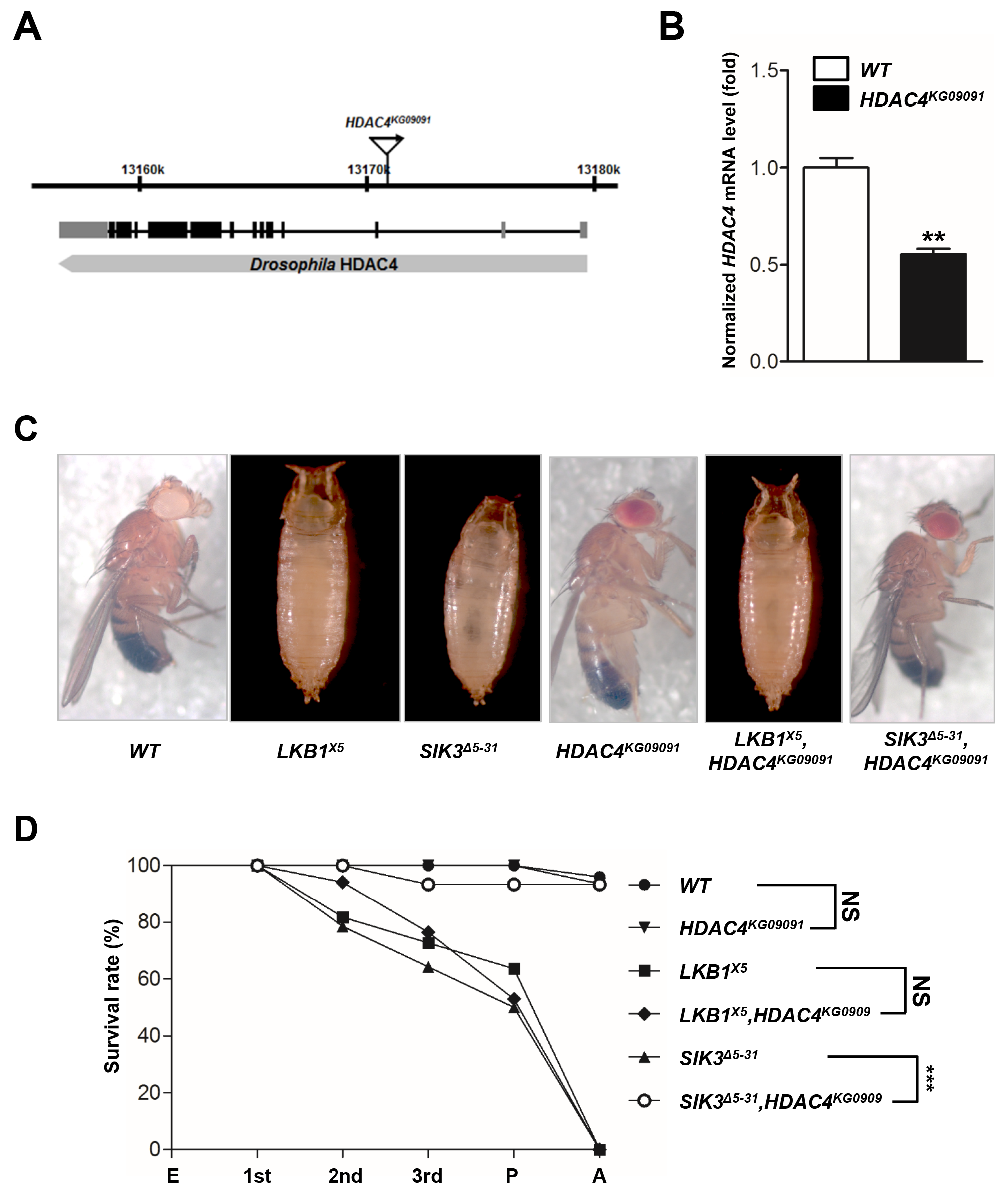

Supplement: S6 Fig — (A) Genomic region of HDAC4 locus. Exons of HDAC4 are indicated by boxes, and coding regions are colored black. The region for HDAC4 hypomorphic mutants (HDAC4 KG09091) is presented. (B) qPCR analysis of HDAC4 mRNA levels in wild-type and HDAC4 mutant adult flies. Data are presented as mean ± SEM (**P < 0.01). (C-D) Effects of HDAC4 gene disruption on fly development (C) and survival rates (D) in LKB1 and SIK3 mutants. Experimental and control survival rates are compared using the log-rank test (***P < 0.001; NS, non-significant). Genotypes are as follows: WT (w 1118), LKB1X5 (LKB1 X5/LKB1 X5), SIK3Δ5–31 (SIK3 Δ5–31 /SIK3 Δ5–31), HDAC4KG09091 (HDAC4 KG09091), LKB1X5,HDAC4KG09091 (HDAC4 KG09091 ;;LKB1 X5/LKB1 X5), and SIK3Δ5–31,HDAC4KG09091 (HDAC4 KG09091 ;SIK3 Δ5–31 /SIK3 Δ5–31). (TIF) [file pgen.1005263.s006.tif]

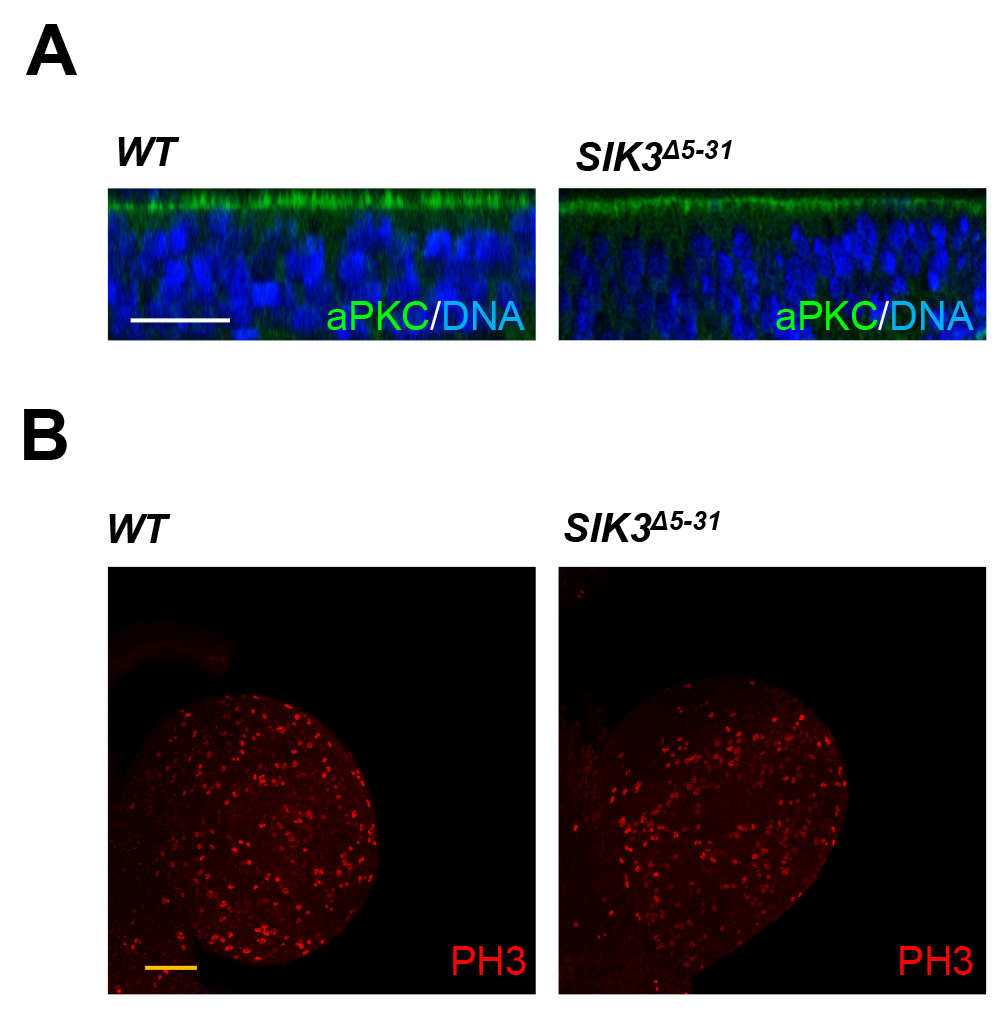

Supplement: S7 Fig — Wing discs (A) and brain hemispheres (B) in wild-type and SIK3 null mutant larvae stained with anti-aPKC (apical complex marker, green) antibody and Hoechst 33258 (DNA, blue) (A), or with anti-PH3 antibody (B). Vertical images were obtained from the Z-stack. Scale bars, white, 10 mm; yellow, 50 mm. Genotypes are as follows: WT (w 1118) and SIK3Δ5–31 (SIK3 Δ5–31 /SIK3 Δ5–31). (TIF) [file pgen.1005263.s007.tif]

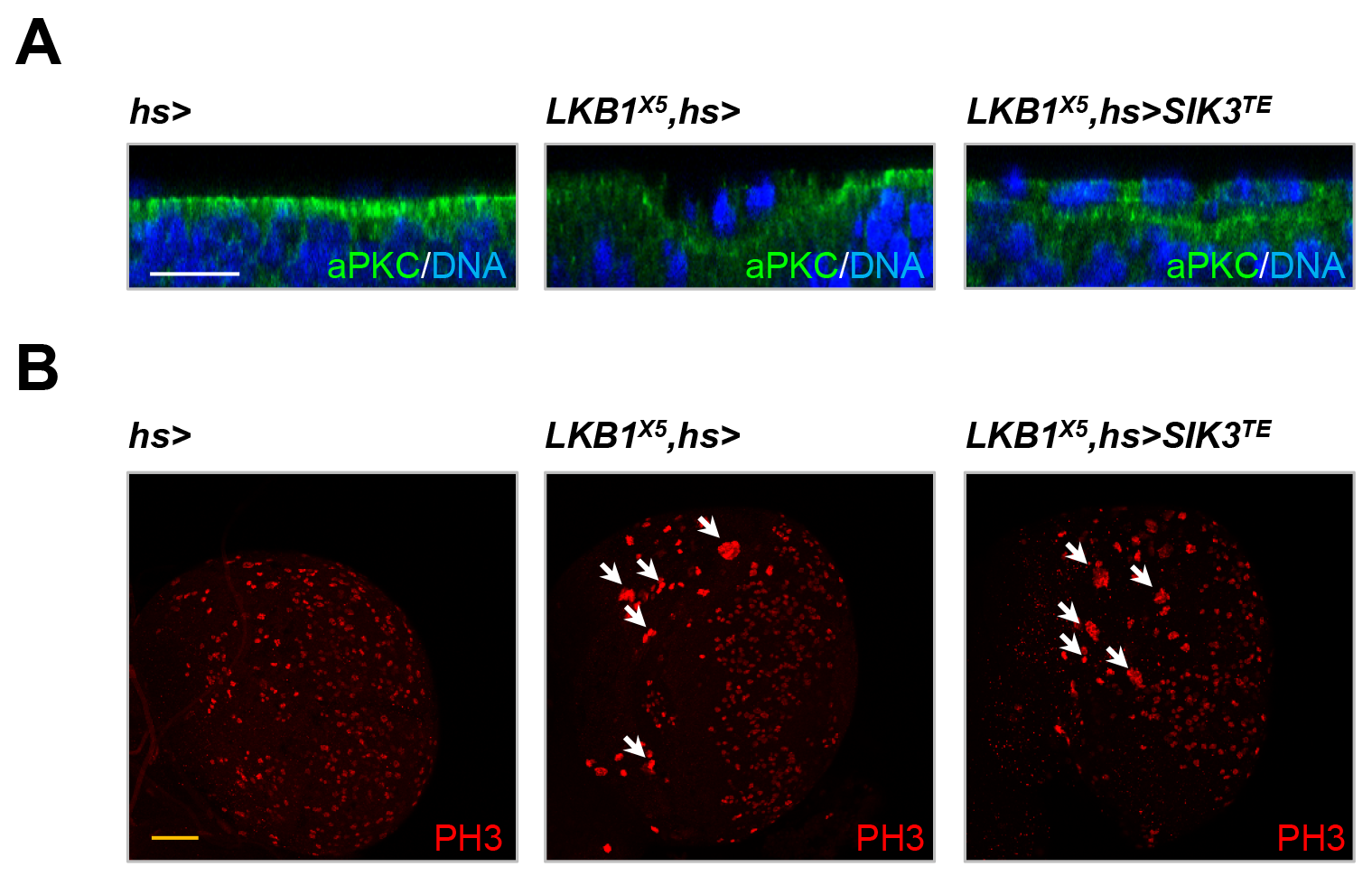

Supplement: S8 Fig — Wing discs (A) and brain hemispheres (B) in wild-type, LKB1 null mutant, and LKB1 null expressing SIK3TE (LKB1X5, hs (heat shock)>SIK3 TE) larvae stained with anti-aPKC (apical complex marker, green) antibody and Hoechst 33258 (DNA, blue) (A), or with anti-PH3 antibody (B). Vertical images were obtained from the Z-stack. The white arrows indicate mitotic chromosomes with polyploidy. Scale bars, white, 10 mm; yellow, 50 mm. Genotypes are as follows: hs> (hs-Gal4/+), LKB1X5,hs> (hs-Gal4/+;LKB1 X5/LKB1 X5), and LKB1X5,hs>SIK3TE (hs-Gal4/UAS-SIK3 T196E;LKB1 X5/LKB1 X5). (TIF) [file pgen.1005263.s008.tif]
