# Supplementary material for: Feeding and Fasting Signals Converge on the LKB1-SIK3 Pathway to Regulate Lipid Metabolism in Drosophila
Source: PLoS Genet. 2015 May 21;11(5):e1005263. doi: 10.1371/journal.pgen.1005263 (PMC4440640; doi:10.1371/journal.pgen.1005263)
Supplement: S1 Table — (DOCX) [file pgen.1005263.s009.docx]

| Gene | Forward | Reverse |
| --- | --- | --- |
| HDAC4(CG1770) | 5’-ATGGCCATGGTATCTACGCT-3’ | 5’-ACCACATTCAGTTGCACAGC-3’ |
| LKB1(CG9374) | 5’-GCCGTCAAGATCCTGACTA-3’ | 5’-CTCCGCTGGACCAGATG-3’ |
| STRAD(CG40293) | 5’-GGCATGATTGGAACGGTTTA-3’ | 5’-TATGTTGAAGCCGACGAACA-3’ |
| MO25(CG4083) | 5’-CGATTTCGAGGGCAAGAA-3’ | 5’-TCTCCGGCTCGGATATGT-3’ |
| SIK2(CG4290) | 5’-CTCGCGTCTTGTCCGACCCAATG-3’ | 5’-GTATGCCAGCCAAGGAGAGATCTTCG-3’ |
| SIK3(CG42856) | 5’-CGACCAGCAAGATATCCGTG -3’ | 5’-CTGCGACTCCATCACCTCG-3’ |
| CG15071 | 5’-ATTTTGTGGCCACCGTTAAG-3’ | 5’-GCGTACTGCTCCCTCAGTTC-3’ |
| AMPKα(CG3051) | 5’-GATCACACGCGTCAAGGTGGCC-3’ | 5’-GGTCTCGATGCACGATCATGTGCC-3’ |
| AMPKβ(CG8057) | 5’-GCTGCGGGGGGTGGA-3’ | 5’-GCTTGGGATCGTGCTTCC-3’ |
| AMPKγ(CG17299) | 5’-AGGATGATCCGGATGATGAC-3’ | 5’-TGGAGATGGACGGGAAGG-3’ |
| SREBP(CG8522) | 5’-CGCAGTTTGTCGCCTGATG-3’ | 5’-CAGACTCCTGTCCAAGAGCTGTT-3’ |
| ACC(CG11198) | 5’-GTGCAACTGTTGGCAGATCAGTA-3’ | 5’-TTTCTGATGACGACGCTGGAT-3’ |
| FAS(CG3523) | 5’-GACTTGACCGATCCGATCAAC-3’ | 5’-CCCCAGGAGGTGAACTCTATCA-3’ |
| bmm(CG5295) | 5’-GTCCCTTCAGTCCCTCCTTC-3’ | 5’-TATGAAGCACGCACACAACA -3’ |
| HSL(CG11055) | 5’-GTTCGCATGCGGAAATCACACTGC-3’ | 5’-GAGAACTCCGCGTATCGAGTCG-3’ |
| rp49(CG7939) | 5’-CCCAAGGGTATCGACAACAG-3’ | 5’-CGATGTTGGGCATCAGTACT-3’ |
